# Organic Acids for Lignin and Hemicellulose Extraction from Black Liquor: A Comparative Study in Structure Analysis and Heavy Metal Adsorption Potential

**DOI:** 10.3390/polym18020251

**Published:** 2026-01-16

**Authors:** Patrycja Miros-Kudra, Paulina Sobczak-Tyluś, Agata Jeziorna, Karolina Gzyra-Jagieła, Justyna Wietecha, Maciej Ciepliński

**Affiliations:** 1 Łukasiewicz—Lodz Institute of Technology, M. Sklodowskiej-Curie 19/27, 90-570 Lodz, Poland; paulina.sobczak@lit.lukasiewicz.gov.pl (P.S.-T.); agata.jeziorna@lit.lukasiewicz.gov.pl (A.J.); karolina.gzyra-jagiela@lit.lukasiewicz.gov.pl (K.G.-J.); justyna.wietecha@lit.lukasiewicz.gov.pl (J.W.); maciej.cieplinski@lit.lukasiewicz.gov.pl (M.C.); 2 Institute of General and Ecological Chemistry, Lodz University of Technology, Zeromskiego 116, 90-924 Lodz, Poland

**Keywords:** lignin, hemicellulose, organic acid, adsorption, heavy metal

## Abstract

This study presents a method for extracting lignin and hemicellulose from black liquor using organic acids (citric, malic, and acetic) in comparison to the traditional sulfuric acid method. We investigated and compared the influence of the acid type on the structural properties of the resulting precipitates in the context of their potential applications. The lignin fractions were characterized for their chemical structure (ATR-FTIR, NMR), thermal stability (TGA), morphology and surface elemental composition (SEM-EDS), bulk elemental composition (C, H, N, S), and molecular weight distribution (GPC). The hemicellulose fractions were analyzed for their molecular weight (GPC), surface elemental composition (EDS), and chemical structure (ATR-FTIR). These analyses revealed subtle differences in the properties of the individual materials depending on the extraction method. We showed that organic acids, particularly citric acid, can effectively precipitate lignin with yields comparable to the sulfuric acid method (47–60 g/dm^3^ vs. 50 g/dm^3^). Simultaneously, this method produces lignin with higher purity (regarding sulfur content) and an increased content of carboxyl groups. This latter aspect is of particular interest due to the enhanced potential of lignin’s adsorption functions towards metal ions. AAS analysis confirmed that lignin precipitated with citric acid showed better adsorption efficiency towards heavy metals compared to lignin precipitated with sulfuric acid, especially for Cu^2+^ ions (80% vs. 20%) and Cr^3+^ ions (46% vs. 2%). This enhanced adsorption efficiency of the isolated lignins, combined with the environmental benefits of using organic acids, opens a promising perspective for their application in water treatment and environmental remediation. Furthermore, the presented research on the valorization and reuse of paper industry by-products fully aligns with the fundamental principles of the Circular Economy.

## 1. Introduction

Lignin, the second most abundant natural polymer after cellulose, has gained significant attention in recent years due to its potential applications in various industries and its role in sustainable development [1,2]. As a major component of lignocellulosic biomass, lignin is primarily obtained as a by-product from the pulp and paper industry, with an estimated global production of 50–70 million tons annually. However, only about 2% of this lignin is currently utilized for value-added applications, with the majority being burned for energy recovery [3,4,5,6].

**Scheme 1:**
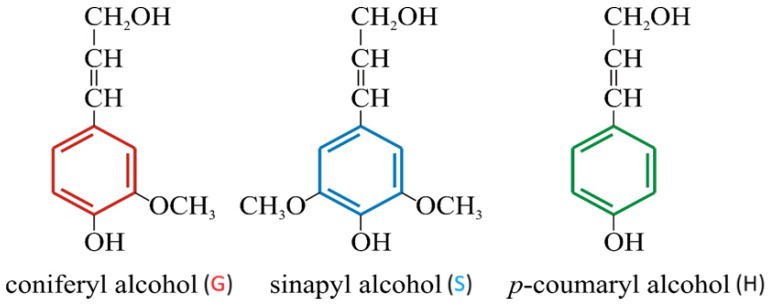
Chemical structures of the three primary lignin monomers.

From a chemical standpoint, lignin is not a discrete macromolecule with a defined primary structure but rather a polymer built from three main lignin monomers: p-coumaryl, coniferyl, and sinapyl alcohols (Scheme 1). These monomers are primarily cross-linked via arylglycerol ether bonds [7].

Technical lignin precipitated from black liquor via acidification exhibits significant variability in its constituent aromatic units, as well as the type and frequency of inter-unit bonds. It is well-established that not only the biomass source but also the mechanical and chemical methods employed for lignin isolation can induce modifications in its native structure. These changes arise from both the cleavage of chemical bonds and the formation of novel structures during the pulping process, particularly under sulfate pulping conditions. Indeed, the sulfate method is characterized by prolonged exposure of wood to elevated pH and temperatures exceeding 160 °C, rendering the black liquor solution an excellent medium for promoting various sulfur-initiated radical processes [8]. Furthermore, technical lignins are often contaminated with carbohydrate compounds.

Besides lignin, hemicelluloses are another important component of lignocellulosic biomass. Hemicelluloses are heteropolysaccharides that, together with cellulose and lignin, form the structural backbone of plant cell walls [9]. Unlike cellulose, hemicelluloses have a branched structure and are composed of various sugar monomers such as xylose, arabinose, mannose and galactose [10]. Potential applications of hemicelluloses include, among others, the production of biofuels, ingredients for the food industry, pharmaceutical and medical applications, advanced biomaterials and composites, and animal nutrition [11,12,13,14].

Traditionally, the kraft process, which uses sodium hydroxide and sodium sulfide for wood delignification, has been the dominant method in the pulp and paper industry [15]. The waste remaining from the process, so-called black liquor, contains dissolved lignin, which is usually precipitated using sulfuric acid [16]. However, this method has raised concerns about its environmental impact due to the use of strong mineral acids and the potential release of harmful sulphur compounds [17,18].

In recent years, there has been an increasing emphasis on developing more sustainable and environmentally friendly methods for the extraction of lignin and hemicellulose [16,19,20,21,22,23,24]. Organic acids have emerged as a promising alternative to sulfuric acid, offering a number of advantages, such as reduced destructive impact on the environment and the production of “greener” lignin and hemicellulose products [25,26,27]. Furthermore, their role often extends beyond being merely milder acids. Citric acid, in particular, is a well-documented natural chelating agent, capable of complexing a wide range of heavy metal ions, including Cu, Cr, and Ni, due to its carboxyl groups acting as ligands [28]. This inherent metal-binding ability leads to the hypothesis that using citric acid as a precipitant may induce surface modification of lignin (e.g., via esterification), thereby increasing the content of carboxyl groups and enhancing its adsorption capacity for heavy metal ions.

The application of lignin as a adsorbent for heavy metal removal addresses a critical environmental challenge, given that water contamination poses significant risks to ecosystems and human health due to the metals’ toxicity, persistence, and bioaccumulation tendency [29,30,31,32,33,34,35,36]. Therefore, the primary objective of this study is to engineer functional lignin materials from industrial black liquor by strategically selecting the precipitation agent. We aim to determine how replacing traditional sulfuric acid with organic acids (citric, malic, and acetic) influences the material’s structural, thermal, and morphological characteristics, and consequently, its adsorption efficiency for Zn, Cu, Ni, and Cr ions. Crucially, the co-precipitated hemicellulose fraction is also characterized to provide a complete assessment of the valorization process.

## 2. Materials and Methods

### 2.1. Materials

Black liquor from the softwood kraft pulping process (20.20% dry matter content, pH 13.07) was used as the primary raw material. All chemicals were of analytical grade and used as received without further purification.

Lignin was precipitated from the black liquor using sulfuric acid (H_2_SO_4_, 95–98%, Chempur, Piekary Śląskie, Poland; sample code: LS), acetic acid (CH_3_COOH, 80%, Chempur, Piekary Śląskie, Poland; LA), citric acid (C_6_H_8_O_7_, PolAura, Dywity, Poland; LC), and malic acid (C_4_H_6_O_5_, PolAura, Dywity, Poland; LM). Hemicelluloses were precipitated with ethyl alcohol (C_2_H_5_OH, 96%, Chempur, Piekary Śląskie, Poland) yielding fractions denoted as HS, HA, HC, and HM, respectively. The metal salts used for adsorption experiments included Cr(NO_3_)_3_·9H_2_O, Ni(NO_3_)_2_·6H_2_O, Zn(NO_3_)_2_·6H_2_O, and Cu(NO_3_)_2_·3H_2_O (all from POCH S.A., Gliwice, Poland).

### 2.2. Lignin and Hemicellulose Extraction

Lignin fractions were isolated from black liquor, a by-product of the kraft pulping of softwood. Lignin was precipitated using organic acids—citric, malic, and acetic. Depending on the strength of the acid used, it was added to 250 mL of black liquor until a stable pH between 4.3 and 5.3 was achieved (Table 1). The target pH values for precipitation were not selected arbitrarily but were carefully established based on a combination of literature data and preliminary experiments. The objective was to evaluate the process by maximizing the lignin precipitation yield while minimizing both reagent consumption and potential structural degradation. The different volumes of each acid required to reach the target pH range are a direct consequence of their distinct acid strengths (pKa values). For comparison, the conventional kraft method [27,34] which uses 0.5 M sulfuric acid to adjust the pH to 2.0, is known to yield lignin of high purity [37].

Following the acidification step, the precipitated lignin was separated by centrifugation. To purify the material and remove non-lignin residues, a double-precipitation technique was employed: the crude precipitate was redissolved in 0.05 M NaOH and subsequently reprecipitated using the respective acid. The purified lignin was then recovered, washed thoroughly with 0.05 M acid solution followed by distilled water, and dried at 50 °C.

The supernatant collected after the initial lignin precipitation was processed to recover the hemicellulose fraction. The liquid was first concentrated using a rotary evaporator at 55 °C. Hemicelluloses were then precipitated from the suspension by adding 96% ethanol in a 4:1 (*v*/*v*) ethanol-to-water ratio. The resulting solid was filtered using a glass filter (0.4 μm pore size) and subjected to a sequential washing process with ethanol, methanol, and methyl tert-butyl ether to remove residual monomeric carbohydrates. The final hemicellulose product was air-dried at room temperature.

### 2.3. Characterization Methods

#### 2.3.1. Gel Permeation Chromatography (GPC)

Molecular-level analysis of the samples was performed using gel permeation chromatography (GPC/SEC) to determine the molar mass distribution (MMD), average molar masses (Mn, Mw), and polydispersity (Mw/Mn). The GPC/SEC analysis was conducted using an Agilent Technologies 1260 Iso Pump system (Agilent Technologies, Santa Clara, CA, USA) equipped with a 1260 ALS autosampler and an Optilab T-rEX refractive index detector (Wyatt Technology, Santa Barbara, CA, USA).

Samples were dissolved in 0.5% LiCl in N,N-dimethylacetamide (DMAc) and analysed at a flow rate of 0.5 mL/min. The injection volume was 100 μL. Macromolecular separation was achieved using a set of three PLgel Mixed-A columns (300 mm length, Agilent Technologies, Santa Clara, CA, USA) maintained at 80 °C. The system was calibrated using polystyrene standards.

MMD, Mn, Mw, and Mw/Mn were calculated using conventional calibration methods, with results expressed in units relative to the calibration standards.

#### 2.3.2. Fourier-Transform Infrared Spectroscopy (FTIR)

Fourier-Transform Infrared (FTIR) spectroscopy was performed using a Nicolet iS50 spectrometer (Thermo Fisher Scientific, Waltham, MA, USA) equipped with an attenuated total reflectance (ATR) accessory (Thermo Fisher Scientific, Waltham, MA, USA). The spectra were recorded in the mid-infrared region from 4000 to 400 cm^−1^. Each spectrum was obtained by co-adding 32 scans for both the background and sample at a spectral resolution of 4000 cm^−1^. Other key instrument settings included a scanning speed of 0.4747 cm/s, an aperture of 85.00, a gain of 4.0, and a sensitivity of 80.

#### 2.3.3. Thermogravimetric Analysis (TGA)

Thermogravimetric analysis was performed using a LabsysEvo 1150 TG apparatus (Setaram Instrumentation, Caluire, France) equipped with Calisto data acquisition and analysis software (version 1.2, Setaram Instrumentation, Caluire, France). The instrument measures the mass of a substance as a function of temperature under controlled atmospheric conditions.

Samples were heated from room temperature to 600 °C at a rate of 5 °C/min under a nitrogen atmosphere. The nitrogen flow rate was maintained at 50 mL/min throughout the analysis. Sample masses of approximately 10 mg were used for each measurement.

#### 2.3.4. Elemental Analysis and Carboxyl Group Content

Carbon, hydrogen, nitrogen, and sulfur content were determined using a Vario Macro Cube elemental analyzer (Elementar Analysensysteme GmbH, Langenselbold, Germany). Samples approx. 15 mg were sealed in tin foil and combusted at 1150 °C in the combustion tube and 850 °C in the reduction tube. Gaseous combustion products were purified, selectively separated in absorption columns, and sequentially detected using a thermal conductivity detector (TCD).

Carboxyl group content in lignin was determined according to the USP 38 NF33 method. The sample (1 g dry weight) was dissolved in 0.5 N NaOH solution and titrated with 0.1 N HCl using phenolphthalein as an indicator. A TitroLine 96 automatic potentiometric titrator (SI Analytics, Mainz, Germany) was used for lignin samples.

#### 2.3.5. Nuclear Magnetic Resonance (NMR) Spectroscopy

2D HSQC NMR spectra were recorded in HSQC experiments on the Bruker Avance 400 MHz spectrometer (Bruker BioSpin, Billerica, MA, USA) at 25 °C in DMSO-d6 as the solvent. Data processing was performed using standard Bruker Topspin-NMR software (version 3.5, Bruker BioSpin, Billerica, MA, USA). The central solvent (DMSO) peak was used as an internal chemical shift reference point (δC/δH 39.5/2.49 ppm).

#### 2.3.6. Scanning Electron Microscopy and Energy Dispersive X-Ray Spectroscopy (SEM/EDS)

SEM was performed using a Thermo Scientific Phenom ProX G6 Desktop (Thermo Fisher Scientific, Waltham, MA, USA). All samples were sputtered with a nanometric layer of gold with rotary pumped coater: Quorum -Q150RS (Quorum Technologies Ltd., Laughton, UK). Microscopic analysis parameters: Detection mode: BSD, Operating voltage: 10 kV, Vacuum: 1 Pa, Magnification: 1500×.

Additionally, elemental composition analysis was carried out using the integrated Energy Dispersive X-ray Spectroscopy (EDS) detector (Thermo Fisher Scientific, Waltham, MA, USA). Microscopic analysis parameters: Detection mode: BSD, Operating voltage: 15 kV, Magnification: 1000×.

### 2.4. Heavy Metal Adsorption Experiments

#### 2.4.1. Adsorption Procedure

Adsorption experiments were conducted to evaluate the heavy metal removal capacity of the extracted lignins by determining the adsorption efficiency (%) and adsorption capacity (q_e_, mg/g). Aqueous solutions were prepared by dissolving the respective analytical grade nitrate salts in deionized water to achieve a final metal cation concentration of 10 mg/L. The specific salts used and the native pH values of their solutions were: Zn(NO_3_)_2_·6H_2_O (pH 6.7), Cu(NO_3_)_2_·3H_2_O (pH 4.9), Ni(NO_3_)_2_·6H_2_O (pH 7.0), and Cr(NO_3_)_3_·9H_2_O (pH 4.1).

The adsorption studies were performed by placing approximately 1 g of lignin in 250 mL Erlenmeyer flasks, followed by the addition of 100 mL of the prepared aqueous metal ion solution. Importantly, no external pH adjustment was applied; the experiments were conducted at the native pH of the salt solutions, which falls within the range of approximately 4–7 due to cation hydrolysis. The flasks were sealed with glass stoppers and incubated at a constant temperature of 25 °C for 120 min, with intermittent mixing every 15 min. After the designated contact time, the mixture was filtered under reduced pressure using a WHATMAN 3 filtration (Whatman, Cytiva, Marlborough, MA, USA) set with MUNKTELL filter papers (Ahlstrom-Munksjö, Falun, Sweden). The resulting filtrate was subjected to analysis using Atomic Absorption Spectrometry (AAS) to determine the residual metal ion concentration.

#### 2.4.2. Metal Ion Concentration Analysis (AAS)

Atomic Absorption Spectrometry (AAS) was employed to quantify the residual metal ion concentrations in the filtrates obtained from the adsorption experiments. The analysis was performed using a ContrAA^®^ 700 spectrometer (Analytik Jena GmbH, Jena, Germany).

The wavelengths used for the determination of specific metals were as follows: 213.9 nm for Zn, 324.8 nm for Cu, 232.0 nm for Ni, and 357.9 nm for Cr. Calibration was carried out using standard solutions with concentrations ranging from 0 to 10 mg/L for each metal. The detection limits were 0.005 mg/L, 0.012 mg/L, 0.025 mg/L, 0.034 mg/L, respectively.

The removal efficiency (R, %) and the adsorption capacity (q_e_, mg/g) were calculated using Equations (1) and (2), respectively:

R (%) = (C_0_ − C_e_)/C_0_ × 100% (1)

q_e_ = (C_0_ − C_e_) × V/m (2)

where

- C_0_ and C_e_ are the initial and equilibrium concentrations (mg/L) of metal ions in the solution, respectively;
- V is the volume of the solution (L);
- m is the mass of the adsorbent (g).

## 3. Results and Discussion

### 3.1. Lignin Extraction

**Figure 1:**
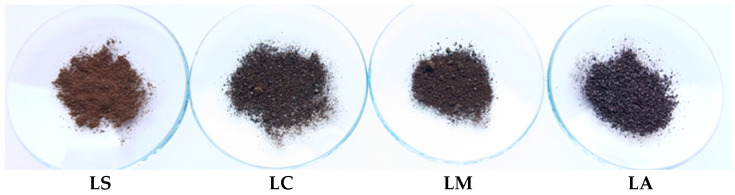
Visual appearance of lignin fractions precipitated using different acids.

The isolation of lignin from industrial black liquor using acids of varying strengths resulted in fractions with pronounced color differences (Figure 1). Precipitation with a strong inorganic acid (sulfuric acid) yielded a light-brown lignin, whereas the use of weaker organic acids (citric, malic, and acetic) consistently produced darker-colored products.

This phenomenon is attributed to the fractional precipitation of lignin based on molecular structure, a well-documented mechanism [38]. It has been demonstrated that precipitation at higher pH values selectively isolates lignin macromolecules characterized by higher molecular weight and a greater degree of condensation. These condensed aromatic structures enlarge the system of conjugated double bonds, which intensifies the absorption of visible light and thus results in a darker material [38].

Consequently, the use of weaker organic acids, which induce precipitation at a relatively high pH, favors the separation of these darker, highly condensed fractions. In contrast, the rapid and strong acidification achieved with sulfuric acid leads to a more comprehensive precipitation of all lignin fractions, including the lighter-colored, less condensed molecules, thereby yielding a product with a lighter overall coloration.

In the pursuit of a more economical and ecological alternative to sulfuric acid, the use of organic acids for lignin precipitation from black liquor was investigated. The process involved adding 0.5 M solutions of organic acids (malic, citric, and acetic) until the pH stabilized within the range of 4.3–5.3. The results, including acid consumption, final pH, and process yield, are presented in Table 1.

This approach yielded high efficiency while limiting reagent consumption. For citric acid, the addition of 400 mL of its solution resulted in a yield of 15.6 g from 250 mL of black liquor. In the case of malic acid, the consumption was 450 mL, with a yield of 14.8 g. Acetic acid, being a weaker acid, required a larger volume (650 mL), which translated to a lower yield (11.6 g).

A comparison of the results (Table 1) demonstrated that organic acids can serve as an effective alternative to the conventional method using sulfuric acid. Citric and malic acids enabled yields comparable to, or even higher than, that obtained with sulfuric acid (13.5 g). Although acetic acid resulted in a lower yield, its application is still relevant within the context of a more sustainable process.

### 3.2. Characterization of Lignins

#### 3.2.1. Gel Permeation Chromatography (GPC) Analysis

**Figure 2:**
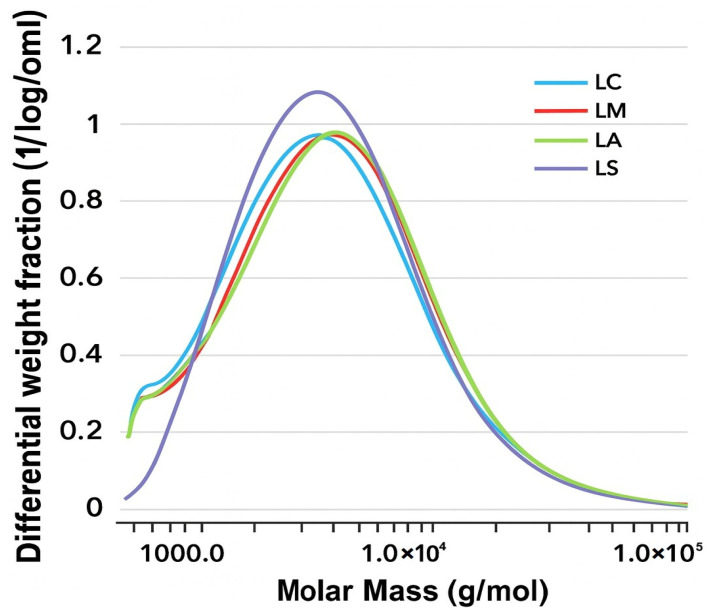
Molecular weight distribution of lignin.

Gel permeation chromatography (GPC) was utilized to assess the molecular weight characteristics of the isolated lignin fractions. The resulting molecular weight distribution (MWD) profiles, depicted in Figure 2, reveal that the choice of precipitating acid fundamentally dictates the final macromolecular structure of the lignin.

While all samples display the broad, unimodal distributions characteristic of lignin’s inherent heterogeneity, a key distinction is observed in the low-molecular-weight region. The lignins precipitated using organic acids (LA, LC, and LM) consistently exhibit a distinct shoulder at approximately 1000 g/mol. The formation of such low-molecular-weight fractions is a well-documented indicator of lignin depolymerization through the cleavage of interunit linkages, primarily the labile β-O-4 ether bonds [39]. This suggests that the weaker organic acids facilitate a controlled depolymerization, yielding a population of stable oligomeric fragments that are preserved during precipitation.

In stark contrast, this shoulder is conspicuously absent for LS. It catalyzes a more aggressive and extensive hydrolysis, not only cleaving the main polymer backbone but also likely breaking down the newly formed oligomers into even smaller fragments.

The quantitative data summarized in Table 2 corroborate this interpretation. Notably, the LS sample exhibits the lowest weight-average molecular weight (Mw = 6 423 g/mol), consistent with extensive chain cleavage. Critically, the polydispersity index (PDI), which measures the breadth of the molecular weight distribution, also reveals a fundamental difference. LS has the lowest PDI value of 2.36, indicating a more uniform polymer population with a narrower mass distribution. In contrast, the lignins obtained using organic acids display higher PDI values (up to 2.58), signifying a broader and more varied distribution of molecular weights. This is perfectly aligned with the MWD curves: the controlled depolymerization by organic acids generates a mixed population of large polymers and smaller oligomers (the shoulder), which naturally broadens the overall distribution.

#### 3.2.2. Fourier-Transform Infrared Spectroscopy (FTIR) Analysis

**Figure 3:**
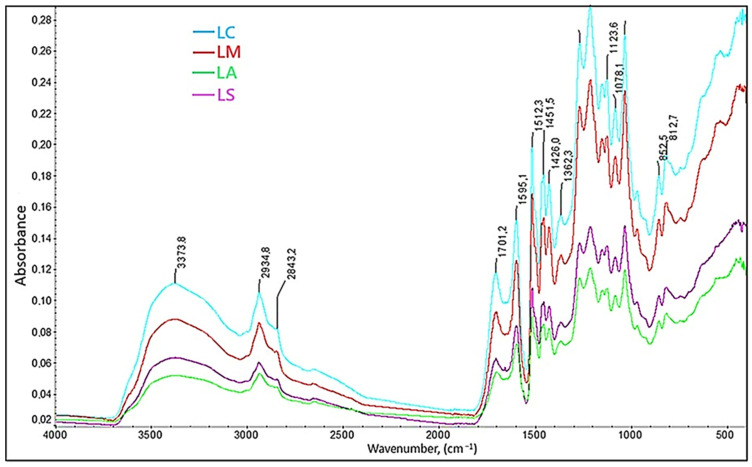
FTIR spectra for precipitated lignin samples.

Fourier-Transform Infrared Spectroscopy with Attenuated Total Reflectance (FTIR-ATR) was employed to characterize the functional groups present in the extracted lignin fractions. This analysis confirmed the successful precipitation of lignin, as evidenced by the presence of characteristic functional groups (Figure 3).

The FTIR spectra of all lignin samples exhibited characteristic absorption bands consistent with typical lignin structures. However, notable variations in band intensities were observed depending on the acid used for precipitation. LC showed the highest absorbance values across the spectrum, particularly for bands associated with O-H (3373 cm^−1^), C-C_arom._ (1595 cm^−1^), and C-O stretching (1264 cm^−1^) vibrations. In contrast, LA exhibited the lowest overall absorbance intensities. These differences in peak intensities suggest subtle variations in the lignin structures, particularly an increase in carboxyl group content, which can be attributed to the specific extraction conditions associated with each acid [40]. Table S1 summarizes the key absorption bands and their corresponding functional groups.

Furthermore, the absence of a characteristic absorption band for the β-(1,4)-glycosidic linkage in cellulose around 898 cm^−1^ across all samples substantiates that the precipitated materials are free from significant cellulose contamination [41].

#### 3.2.3. Elemental Analysis and Carboxyl Group Content

Elemental analysis and carboxyl group content determination were performed on the lignin samples to assess the impact of different precipitation acids on their chemical composition. Table 3 presents the results of this analysis, including the content of carbon, hydrogen, nitrogen, sulfur, and carboxyl groups.

Elemental analysis revealed differences in sulfur content among lignins precipitated with various acids. LS showed the highest sulfur content (5.72%), which is typical for this extraction process because the active functional groups of lignin can partially undergo sulfonation reactions. Lignins precipitated with organic acids exhibited lower sulfur content: 4.18% for LA, 3.33% for LC, and 3.12% for LM. This difference may result from the nature of the acids used and their interactions with lignin during the precipitation process. The carbon content in LS, LM, and LC was relatively consistent (60.1–61.29%), while LM showed a slightly lower carbon content (52.35%). An interesting trend is the increase in carboxyl group content in lignins precipitated with organic acids, particularly noticeable for LC (10.8%) compared to LS (7.4%). This observation may indicate potential modifications to the lignin structure during the precipitation process with organic acids, which could influence its properties and potential applications.

#### 3.2.4. Thermogravimetric (TGA) Analysis

Thermogravimetric analysis was employed to evaluate the thermal stability of the extracted lignin samples. This technique provides crucial information about the thermal degradation behavior of lignin, which is essential for assessing its potential industrial applications. The TGA curves of lignin samples extracted using different acids exhibited distinct degradation profiles (Table 4).

The TGA and DSC curves (Figures S1–S4) revealed similar thermal behavior patterns across all samples. The mass loss process occurred in two main stages:

1. Initial stage: Primarily associated with moisture desorption, resulting in a mass loss of 4.6–5.8%.
2. Main decomposition stage (≥136 °C): Characterized by the thermal decomposition of lignin structures.

The main decomposition process initiated at approximately 136 °C and proceeded in multiple steps. Samples exhibited three distinct mass loss rate maxima, as evidenced by the differential thermogravimetry (dTG) curves (Figures S5–S8).

Notably, LC exhibited high thermal stability with a decomposition temperature of 182 °C, compared to LS, which decomposed at 136 °C. This significant difference in thermal stability highlights the impact of the extraction method on the properties of the obtained lignin.

The DSC curves displayed an endothermic peak below 100 °C for all samples, corresponding to the evaporation of residual moisture. At higher temperatures, the DSC curves showed a clear exothermic trend, indicating heat release during the decomposition process.

The observed variation in the residue yield (char content) correlates with the structural differences of the precipitates. LS exhibited the lowest char yield (60.6%), which can be attributed to the extensive acid-catalyzed depolymerization and lower molecular weight of this fraction (as confirmed by GPC analysis). In contrast, LC showed the highest thermal stability and char residue (65.8%), likely due to a higher degree of condensation and potential cross-linking reactions involving the polycarboxylic acid, which favor the formation of stable aromatic char structures. All lignin samples demonstrated minimal degradation up to approx. 160 °C, indicating good thermal stability in this temperature range.

The mass loss observed between 230–270 °C can be attributed to the dehydration of hydroxyl groups, while the degradation occurring around 340–370 °C likely corresponds to the cleavage of aliphatic side chains from the aromatic rings [25].

It is worth noting that the precise interpretation of lignin decomposition results can be challenging due to the partial or complete overlap of temperature ranges for different stages of the process.

The detailed results of the TG-DSC analysis, including the temperatures of maximum mass loss rates and the onset and end temperatures of the process, are presented in Table 4, and illustrated in the DSC (blue) and dTG (purple) curves (Figures S1–S8).

#### 3.2.5. Nuclear Magnetic Resonance (NMR) Spectroscopy

One of the methods of studying the structural features of lignin is nuclear magnetic resonance spectroscopy, especially heteronuclear two-dimensional NMR spectroscopy (2D NMR).

In our studies, the short-range correlation experiment 1 H–13 C HSQC was used in the qualitative analysis of lignin to assign characteristic units, the structures of which have already been well recognized and described in detail in the literature [42,43,44].

In the 2D HSQC spectra of individual lignins precipitated using different acids, we can see cross peaks of 1 H–13 C nuclei accumulated in 3 areas (Figures S9–S12). The least characteristic area, with an unstable composition, resulting primarily from biomass processing, is the region δH 0.5–2.5 ppm/δC 10–40 ppm, indicating the presence of long hydrocarbon chains present in fatty acids, alkanes (common in waxes) and alkyl parts of various lipids. These low-molecular fragments of lignin are usually products of its degradation, i.e., depolymerization or fragmentation. In our studies for lignins precipitated with different acids, this region is almost identical for all variants, hence it can be assumed that the origin of aliphatic compounds in the tested fraction is primarily the result of the industrial process during the isolation of cellulose from wood (Kraft process). Nevertheless, in the spectrum of lignin precipitated with citric acid, an additional distinct cross peak was observed in the alkyl region (δC/δH: 43/0~2.5 ppm), which may correspond to CH_2_ groups—connected to lignin structures by ether or ester bonds. The appearance of this signal may be evidence that citric acid not only neutralizes the black liquor environment with the precipitation of insoluble lignins, but also enters into chemical reactions with active lignin groups. In the region of oxidized aliphatic groups, a strong signal from methoxyl groups and signals indicating the presence of xylans are visible in all spectra. Differences in this area may result from the attachment of acid residues of organic acids to active lignin groups and in the case of sulfuric acid from the possibility of sulfonation of these functions. The third characteristic region corresponds to unsaturated aliphatic groups and aromatic rings. The spectrum richest in cross-peaks belongs to lignin precipitated with sulfuric acid. Signals in the region δC/δH: ~90–110/4~5 ppm probably correspond to C-H groups bound to sulfonic groups (-SO_3_H), the presence of which was confirmed by elemental analysis (the presence of sulfur).

#### 3.2.6. SEM-EDS Analysis of Lignin Surface Morphology

**Figure 4:**
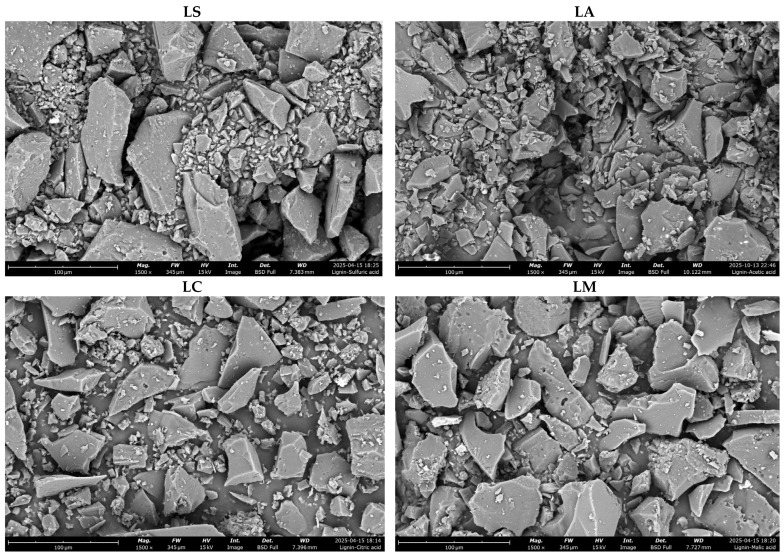
SEM image of lignin products obtained using a scanning electron microscope. Magnification 1500×.

Analysis by scanning electron microscopy (SEM) indicated that all isolated lignin fractions share a similar morphology, consisting of compact agglomerates of irregularly shaped particles (Figure 4). This visual assessment was quantitatively supported by pycnometric density measurements. All samples exhibited low densities (0.0267–0.0336 g/cm^3^). Although variations were observed, these results indicate that the precipitation method consistently yields materials with a loose macroscopic structure.

To complement the morphological observations with elemental data, energy-dispersive x-ray spectroscopy (EDS) analysis was conducted on the initial lignin samples prior to the adsorption experiments. This analysis confirmed the elemental purity of the material, revealing the absence of any metallic contaminants. Following the adsorption process, EDS analysis of the same lignin samples provided direct evidence of successful metal uptake, confirming the presence of the respective adsorbed metals through their characteristic elemental peaks. The comparative spectra are provided in the Supplementary Information (Figures S13–S15).

### 3.3. Heavy Metal Adsorption Performance of Extracted Lignins

Prior to the adsorption experiments, the purity of the initial lignin samples was verified using EDS analysis (as described in Section 3.2.6). The spectra confirmed the absence of any heavy metal contaminants (Ni, Cu, Zn, Cr) in the starting materials, ensuring that the metals detected post-adsorption originated solely from the experimental solution.

**Figure 5:**
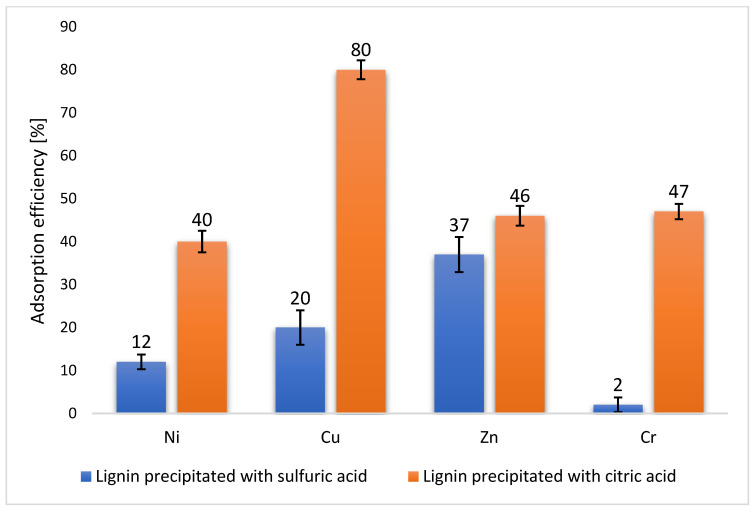
Heavy metal adsorption efficiency (%) of lignins extracted with sulfuric acid and citric acid. Error bars represent the standard deviation of three independent experimental replicates (*n* = 3).

The adsorption efficiency of LS and LC was evaluated for four heavy metal ions: nickel (Ni), copper (Cu), zinc (Zn), and chromium (Cr). The results demonstrate significant differences in adsorption efficiency between the two extraction methods (Figure 5).

While Figure 5 illustrates the removal efficiency, the equilibrium adsorption capacity (q_e_) was also calculated to assess the material’s performance per unit mass (Table 5). It is important to note that under the applied experimental conditions (C_0_ = 10 mg/L and adsorbent dosage of 10 g/L), the theoretical maximum capacity was restricted to 1.0 mg/g by mass balance. Despite this limitation, the q_e_ values reveal a fundamental difference in affinity. LC consistently showed higher capacities compared to LS across all tested ions. The most pronounced improvement was observed for Cu^2+^ (0.80 vs. 0.20 mg/g) and Cr^3+^ (0.47 vs. 0.02 mg/g). This confirms that organic acid precipitation significantly enhances the density of active binding sites available for metal capture, making the material far more effective even at low metal concentrations.

Based on the adsorption efficiency, the affinity of LS for different metal ions followed the order Zn > Cu > Ni > Cr. For LC, the adsorption affinity order was Cu > Zn ≈ Cr > Ni.

The significantly higher adsorption efficiency of LC, particularly for copper (80%) and chromium (46%), can be attributed to several factors. The citric acid extraction process likely alters the lignin’s molecular structure, potentially increasing the number of available binding sites or enhancing its interaction with metal ions. This structural modification is evidenced by the increased carboxyl group content observed in the elemental analysis of citric acid-extracted lignin.

Furthermore, the nature of the metal ions plays a crucial role in the adsorption process. All four metal ions (Ni, Cu, Zn, and Cr) belong to the d-block and 4th period of the periodic table, having similar atomic masses, ionic radii, and electronegativity. However, their interactions with the lignin structures differ, possibly due to variations in coordination preferences and binding strengths. The observed differences in adsorption efficiencies among these metals suggest that the modified lignin structure resulting from citric acid extraction may have a particular affinity for certain metal ions, especially copper and chromium.

### 3.4. Hemicellulose Extraction

**Figure 6:**
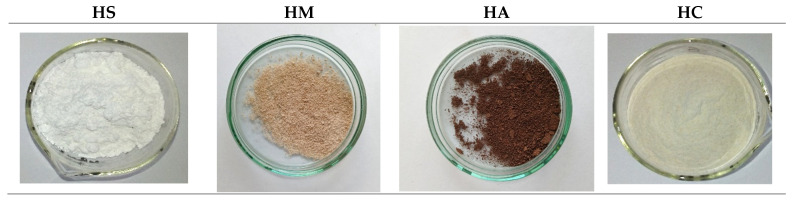
Macroscopic appearance of hemicellulose fractions isolated after lignin precipitation with different acids. The coloration indicates the presence of co-precipitated lignin-derived compounds.

Following the lignin precipitation process described in Section 2.2, hemicelluloses were recovered from the resulting supernatant via ethanol precipitation. The physical appearance and yield of the final hemicellulose products were directly dependent on the type of acid used in the preceding lignin precipitation step (Figure 6 and Table 6).

The hemicellulose fraction isolated from the liquor after lignin precipitation with sulfuric acid was a white powder, indicating high purity. In contrast, hemicelluloses recovered from the liquors where organic acids were used showed varied coloration. The intensity of the color was dependent on the acid used: the powder was light yellow following precipitation with citric acid, slightly brownish with malic acid, while the most intense, dark brown color was observed in the fraction after acetic acid treatment.

### 3.5. Characterization Methods of Hemicellulose

#### 3.5.1. Gel Permeation Chromatography (GPC) Analysis

Gel Permeation Chromatography (GPC) was employed to evaluate the molecular structure of the extracted hemicelluloses by determining their weight-average molecular mass. The analysis revealed distinct differences in the molecular weight distribution of hemicelluloses depending on the acid used for the initial lignin precipitation (Table 7).

**Figure 7:**
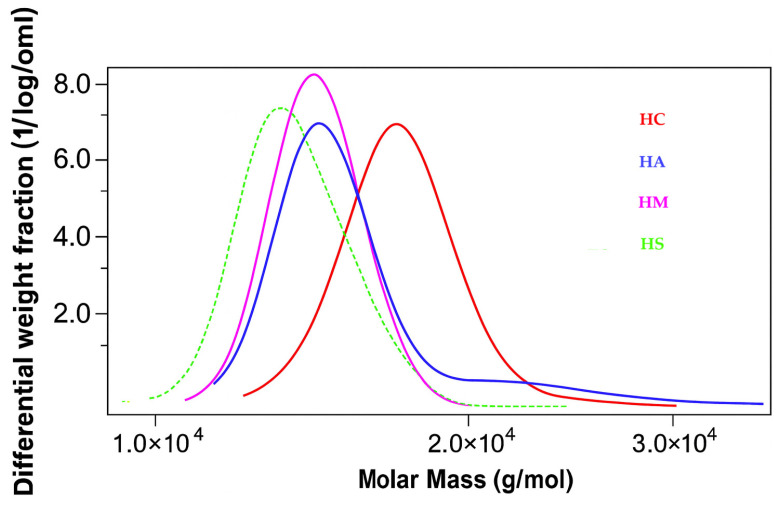
GPC curves showing the molecular weight distribution of hemicelluloses (first peak).

**Figure 8:**
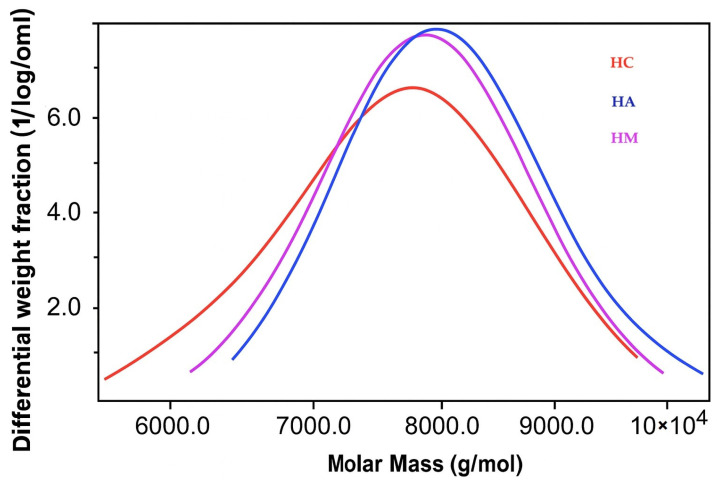
GPC curves showing the molecular weight distribution of hemicelluloses (second peak).

Hemicelluloses isolated from the supernatants of organic acid-based lignin precipitations exhibited a bimodal distribution, characterized by two distinct peaks (Figure 7 and Figure 8):

1. A higher molecular weight fraction with peaks ranging from 14,600 to 17,600 g/mol
2. A lower molecular weight fraction with peaks between 7700 and 8200 g/mol

Both fractions showed a remarkably low polydispersity index (Mw/Mn) of approximately 1.00, indicating highly uniform molecular weight distributions within each fraction.

In contrast, the HS displayed a unimodal distribution with a single peak at approximately 13,700 g/mol.

The bimodal distribution observed in the organic acid pathways may indicate that they are a heterogeneous material, consisting of a mixture of polysaccharides (hemicelluloses) and lignin-derived compounds. This, in turn, explains the observed dark coloration of these samples.

#### 3.5.2. ATR-FTIR Analysis of Hemicellulose Fractions

To elucidate the chemical differences among the hemicellulose fractions, ATR-FTIR spectroscopy was performed (Figures S16–S20).

The spectrum of the HS presents a starkly different profile from the others. It is dominated by a few key signals, primarily intense C-O stretching and C-O-C skeletal vibration bands within the 1200–1000 cm^−1^ fingerprint region, which are fundamental to the polysaccharide backbone. In contrast, the spectra of hemicelluloses obtained from the organic acid routes, while also featuring the core polysaccharide signals, exhibit a series of additional, distinct absorption bands. These bands are unequivocally identified as lignin-derived. Specifically, all three samples (precipitated with citric, malic, and acetic acid) show well-defined peaks corresponding to aromatic skeletal vibrations at 1600, 1595 and 1589 cm^−1^, which serve as a definitive fingerprint of the lignin aromatic core. Furthermore, a prominent signal at ~1259 cm^−1^, characteristic of guaiacyl C-O stretching, is clearly present in these spectra.

This direct spectroscopic comparison provides evidence that the hemicellulose from the sulfuric acid pathway is compositionally purer, consisting mainly of a polysaccharide backbone. In contrast, the hemicelluloses from the organic acid pathways possess, in addition to polysaccharides, bonded lignin-derived compounds, as evidenced by the clear presence of numerous diagnostic lignin bands.

#### 3.5.3. SEM Analysis of Hemicellulose Surface Morphology/EDS Analysis

**Figure 9:**
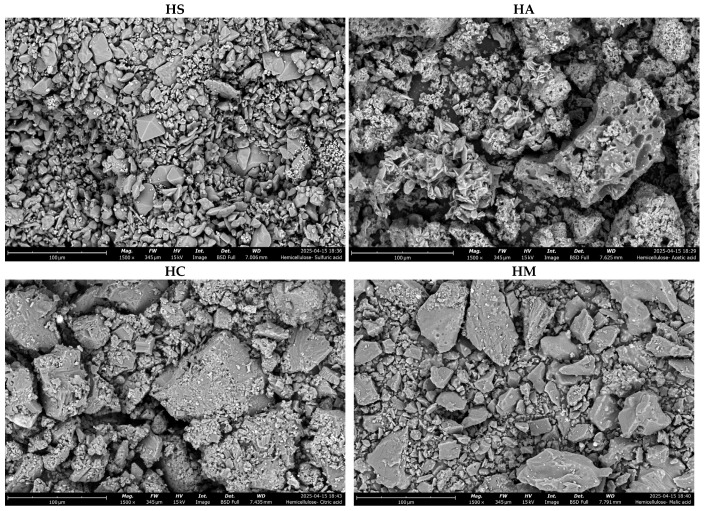
SEM image of hemicellulose products obtained using a scanning electron microscope. Magnification 1500×.

The surface morphology and elemental composition of the hemicelluloses were analyzed using Scanning Electron Microscopy (SEM) and Energy-Dispersive X-ray Spectroscopy (EDS). Hemicelluloses recovered from the supernatants of organic acid-based precipitations consistently exhibited a more porous and irregular surface structure. Conversely, the HS showed a dense, compact, and more uniform morphology (Figure 9).

EDS analysis confirmed fundamental chemical differences between the samples, aligning with findings from both GPC and ATR-FTIR. Hemicelluloses derived from the organic acid pathways (HC, HA, HM) are characterized by a significantly higher atomic concentration of carbon (28.4–31.3%) (Table S2 and Figure S21). This elevated carbon content is consistent with the co-precipitation of lignin-derived compounds, which are rich in aromatic structures. This finding supports the interpretation that these samples are mixtures of polysaccharides and lignin-based impurities. On the contrary, HS exhibited a much lower carbon content (7.8%) but a very high concentration of sulfur (13.2%).

## 4. Conclusions

This study demonstrates that employing organic acids for precipitation is a strategic approach that dictates the structural and functional properties of the resulting materials. A key finding is the trade-off between process outcomes: organic acids yield highly functionalized lignin adsorbents, while the sulfuric acid method ensures high-purity hemicellulose.

Precipitation with citric acid produced lignin with dramatically enhanced heavy metal adsorption capacity, evidenced by efficiency increases from 20% to 80% for Cu^2+^ and from 2% to 46% for Cr^3+^ compared to the sulfuric acid route. This superior performance is directly attributed to structural modifications, specifically higher purity (lower sulfur content) and increased carboxyl group content (10.8% vs. 7.4%) acting as effective metal binding sites.

In contrast, the conventional sulfuric acid method excels in isolating a white, homogeneous hemicellulose fraction with a unimodal molecular weight distribution and no lignin contaminants. Conversely, organic acids yield brownish, mixed lignin–polysaccharide co-precipitates which require further purification.

In conclusion, the choice of acid must be tailored to the targeted application: citric acid is the superior option for designing environmental adsorbents, while sulfuric acid remains more effective for obtaining pure hemicellulose. This study provides a framework for improving biorefinery processes to engineer targeted, high-value products from industrial waste in alignment with the Circular Economy.

## Figures and Tables

**Table 1:** Conditions and yields of lignin precipitation process using various acids.

	Lignin Precipitation		
	Amount of Acid Used [mL]	pH	Process Yield per 250 mL of Black Liquor [g]
LS	460	2.07	13.5
LM	450	4.49	14.8
LC	400	4.32	15.6
LA	650	5.32	11.6

**Table 2:** GPC analysis results of lignin samples expressed in polystyrene standard units. Values represent the average of analysed samples.

Sample Name	Mn [g/mol wg PS ^1^]	Mw [g/mol wg PS]	Mw/Mn
LS	2727	6423	2.36
LM	2690	6950	2.58
LA	3287	8303	2.53
LC	2740	6983	2.55

^1^ PS stands for polystyrene.

**Table 3:** Chemical composition of analyzed lignin samples.

Unit/Element	LS	LM	LA	LC
% C	60.1	52.4	60.9	61.3
% H	5.61	6.25	5.85	5.75
% N	0.08	0.08	0.09	0.09
% S	5.72	3.12	4.18	3.33
% COOH	7.40	9.50	8.80	10.8

**Table 4:** Thermogravimetric characteristics of the lignin samples after precipitation heated at a rate of 5 °C/min under an inert nitrogen atmosphere.

Sample Name	Water Evaporation				Thermal Decomposition						Residue After Measurement
	Δm [%]	T_1_ [°C]	T_inf_ [°C]	T_2_ [°C]	T_1_ [°C]	1	2	3	T_2_ [°C]	Δm [%]	M [%]
LS	4.6	48	82	127	136	156	242	372	401	34.8	60.6
LM	5.5	64	82	107	153	197	248	376	402	31.8	63.0
LA	5.8	49	82	119	165	174	233	342	401	29.9	64.6
LC	5.0	59	94	149	182	197	247	363	402	29.4	65.8

where Δm—mass loss; T_1_—temperature of effective start of mass loss process determined by the company program using the horizontal method; T_inf_—temperature of maximum mass loss process (inflection point on the TG curve corresponding to the peak on the dTG curve); and T_2_—end temperature of measurement. All Δm were determined in relation to the initial sample mass.

**Table 5:** Equilibrium adsorption capacity (q_e_) of lignin samples.

Metal Ion	Lignin Sample	Adsorption Capacity q_e_ * [mg/g]
Ni^2+^	LS	0.12
	LC	0.40
Cu^2+^	LS	0.20
	LC	0.80
Zn^2+^	LS	0.37
	LC	0.46
Cr^3+^	LS	0.02
	LC	0.47

* Calculated based on Equation (2) with initial concentration $C_{0}=10$ mg/L and adsorbent dosage of 10 g/L. The theoretical maximum capacity under these conditions is 1.0 mg/g.

**Table 6:** Yields of hemicellulose precipitation process using various acids.

Sample Name	Process Yield [g/L of Black Liquor]
HS	80.0
HM	43.2
HC	72.0
HA	38.0

**Table 7:** GPC results for hemicellulose samples.

	First Peak			Second Peak		
	Mn [g/mol PS]	Mw [g/mol PS]	Mw/Mn	Mn [g/mol PS]	Mw [g/mol PS]	Mw/Mn
HS	13,600	13,700	1.01	No peak		
HA	15,400	16,100	1.04	8100	8200	1.0
HC	17,300	17,600	1.02	7600	7700	1.0
HM	14,400	14,600	1.01	7900	8000	1.0

## Data Availability

All relevant data are within the paper.

## Supplementary Materials

The following supporting information can be downloaded at: https://www.mdpi.com/article/10.3390/polym18020251/s1, Figure S1: TG (green)–DSC (blue) curves for LC; Figure S2: TG (green)–DSC (blue) curves for LA; Figure S3: TG (green)–DSC (blue) curves for LS; Figure S4: TG (green)–DSC (blue) curves for LM; Figure S5: dTG curve test for LC; Figure S6: dTG curve test for LA; Figure S7: dTG curve test for LS; Figure S8: dTG curve test for LM; Figure S9: NMR spectrum of lignin LS; Figure S10: NMR spectrum of lignin LA; Figure S11: NMR spectrum of lignin LM; Figure S12: NMR spectrum of lignin LC; Figure S13: Elemental composition of lignin samples prior to adsorption, determined by Energy-Dispersive X-ray Spectroscopy (EDS). Spectra and quantitative results (atomic and weight concentration) are presented for lignins precipitated with citric (LC), acetic (LA), malic (LM), and sulfuric (LS) acids; Figure S14: EDS analysis confirming the presence of heavy metals on citric acid lignin (LC) after the adsorption process; Figure S15: EDS analysis confirming the presence of heavy metals on sulfuric acid lignin (LS) after the adsorption process; Figure S16: ATR spectrum of hemicellulose samples; Figure S17: ATR spectrum of HA; Figure S18: ATR spectrum of HC; Figure S19: ATR spectrum of HM; Figure S20: ATR spectrum of HS; Figure S21: Photos obtained using the EDS method of hemicellulose samples; Table S1: Characteristic FTIR absorption bands of lignin samples; Table S2: EDS results for hemicellulose samples.
