# Supplementary material for: Organic Acids for Lignin and Hemicellulose Extraction from Black Liquor: A Comparative Study in Structure Analysis and Heavy Metal Adsorption Potential
Source: Polymers (Basel). 2026 Jan 16;18(2):251. doi: 10.3390/polym18020251 (PMC12845765; doi:10.3390/polym18020251)
Supplement: Supplementary file 1 [file polymers-18-00251-s001.zip › polymers-4049078-supplementary.pdf]

Supplementary Information

Organic Acids for Lignin and Hemicellulose Extraction from Black Liquor: A Comparative Study in Structure Analysis and Heavy Metal Adsorption Potential

Patrycja Miros-Kudra [1,*], Paulina Sobczak-Tyluś [1,2], Agata Jeziorna [1], Karolina Gzyra-Jagieła [1], Justyna Wietecha [1] and Maciej Ciepliński [1]

[1] Łukasiewicz—Lodz Institute of Technology, M. Sklodowskiej-Curie 19/27, 90-570 Lodz, Poland; paulina.sobczak@lit.lukasiewicz.gov.pl (P.S.-T.); agata.jeziorna@lit.lukasiewicz.gov.pl (A.J.); karolina.gzyra-jagiela@lit.lukasiewicz.gov.pl (K.G.-J.); justyna.wietecha@lit.lukasiewicz.gov.pl (J.W.); maciej.cieplinski@lit.lukasiewicz.gov.pl (M.C.)
[2] Institute of General and Ecological Chemistry, Lodz University of Technology, Zeromskiego 116, 90-924 Lodz, Poland
* Correspondence: patrycja.miros-kudra@lit.lukasiewicz.gov.pl

Table S1. Characteristic FTIR absorption bands of lignin samples.

Wave number (cm^{-1})	Assignment
3373	O-H stretching (hydroxyl groups)
2934	C-H stretching in methyl and methylene groups
2843	C-H stretching in methoxyl groups
1701	C=O stretching (carbonyl groups)
1595	Aromatic ring vibrations
1512	Aromatic skeletal vibrations
1450	C-H deformations (asymmetric in methyl and methylene groups)
1426	Aromatic ring vibrations
1362	Phenolic O-H bending
1264	C-O stretching in guaiacyl units
1209	C-O stretching in phenols and aryl ethers
1147	Aromatic C-H in-plane deformation (guaiacyl type)
1123	Aromatic C-H in-plane deformation (syringyl type)
1078	C-O deformation in secondary alcohols and aliphatic ethers

852	C-H out-of-plane deformations in positions 2, 5, and 6 of guaiacyl units
812	C-H out-of-plane deformations in positions 2 and 6 of syringyl units

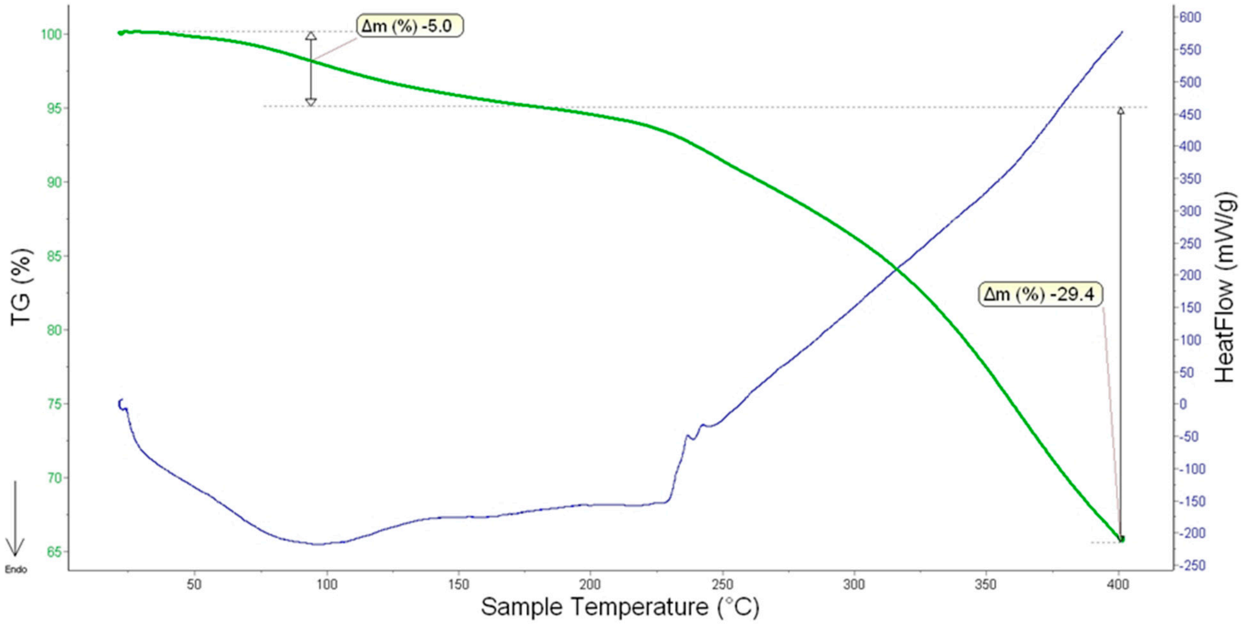

Figure S1. TG (green) - DSC (blue) curves for LC.

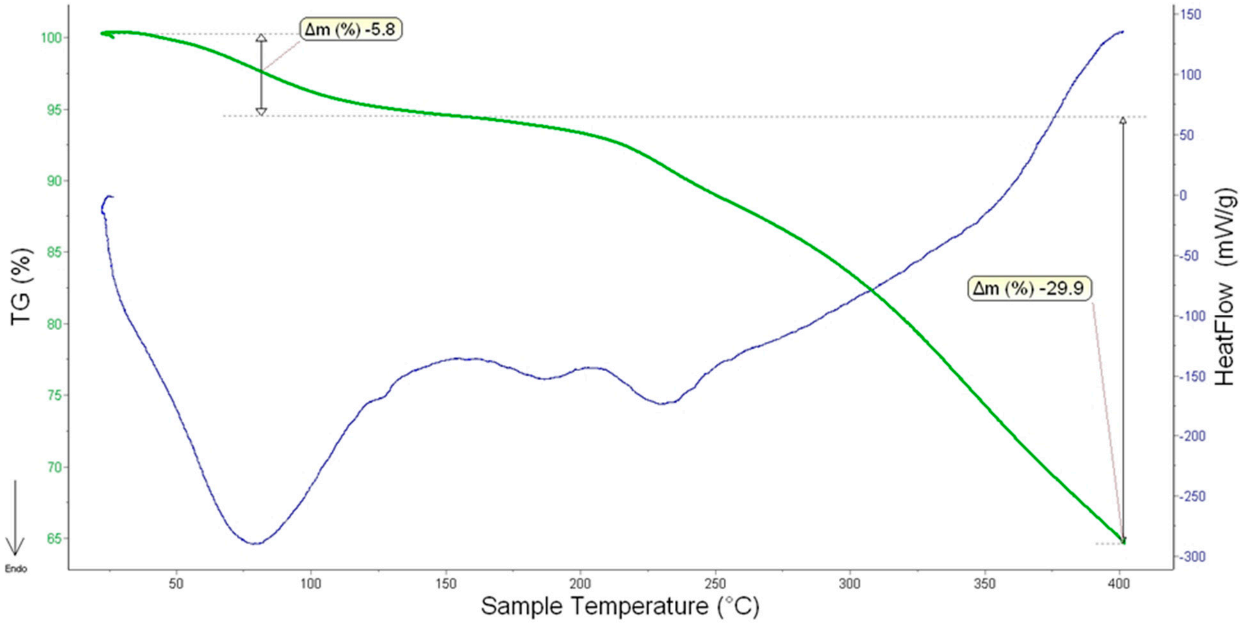

Figure S2. TG (green) - DSC (blue) curves for LA.

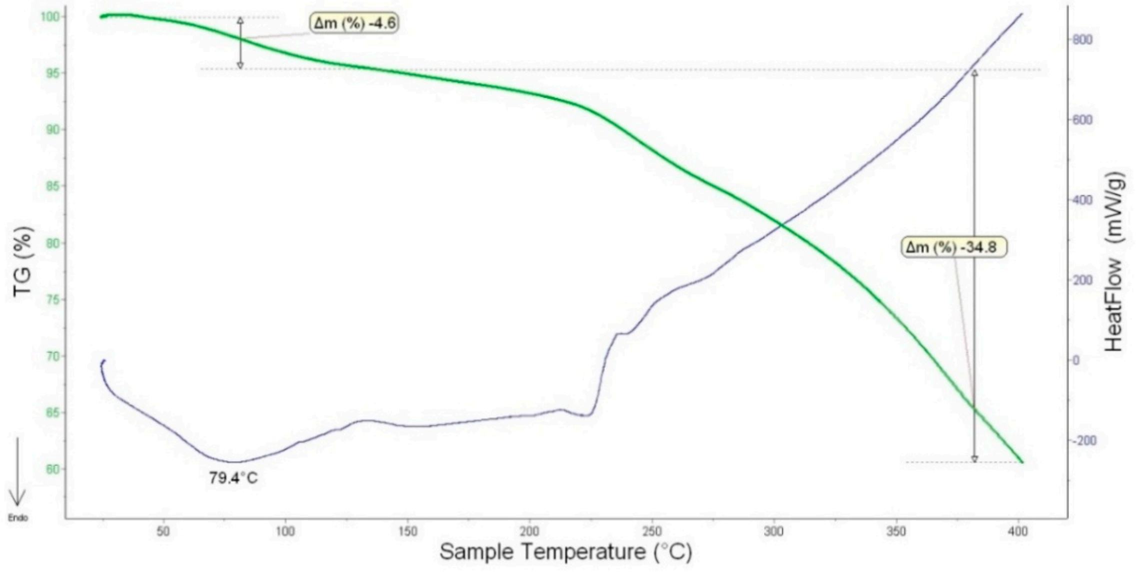

Figure S3. TG (green) - DSC (blue) curves for LS.

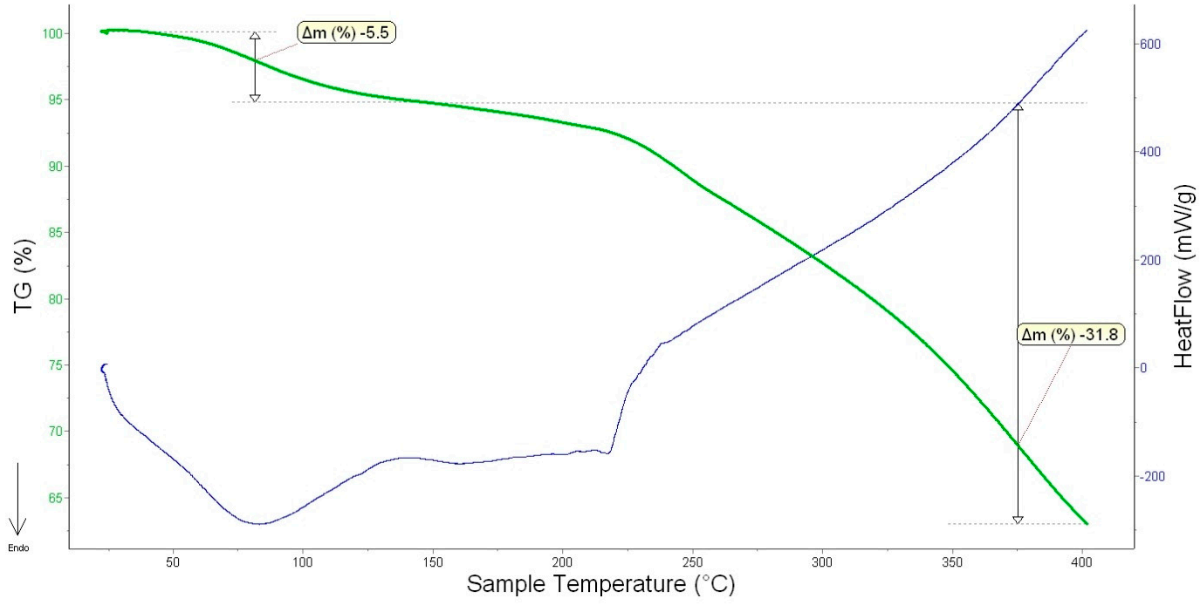

Figure S4. TG (green) – DSC (blue) curves for LM.

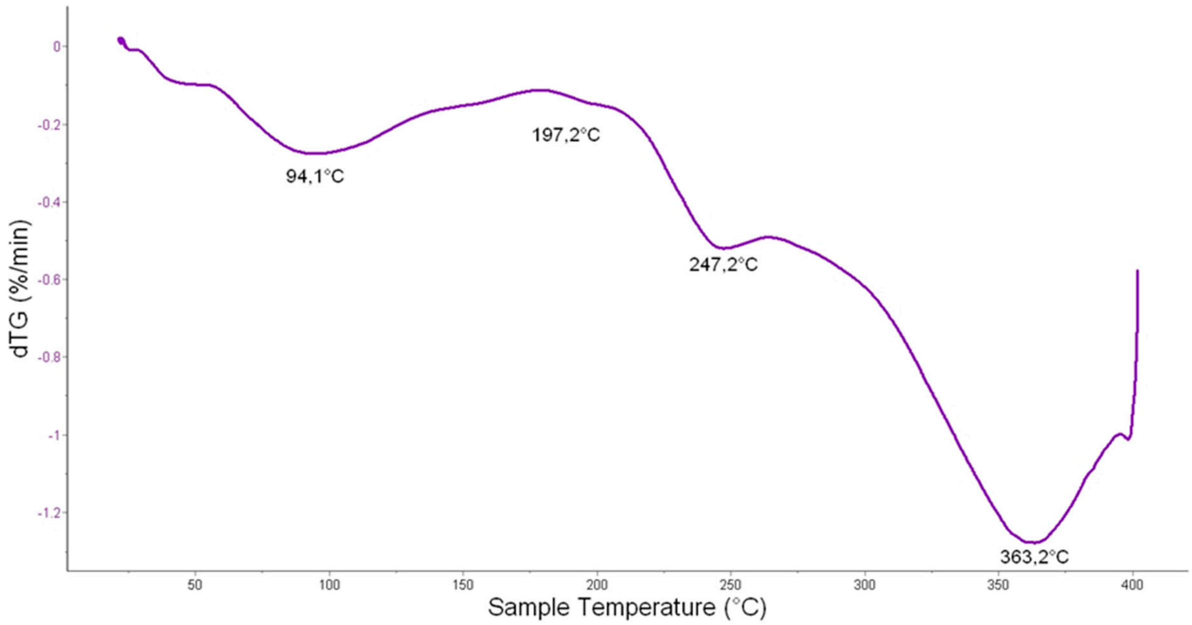

Figure S5. dTG curve test for LC.

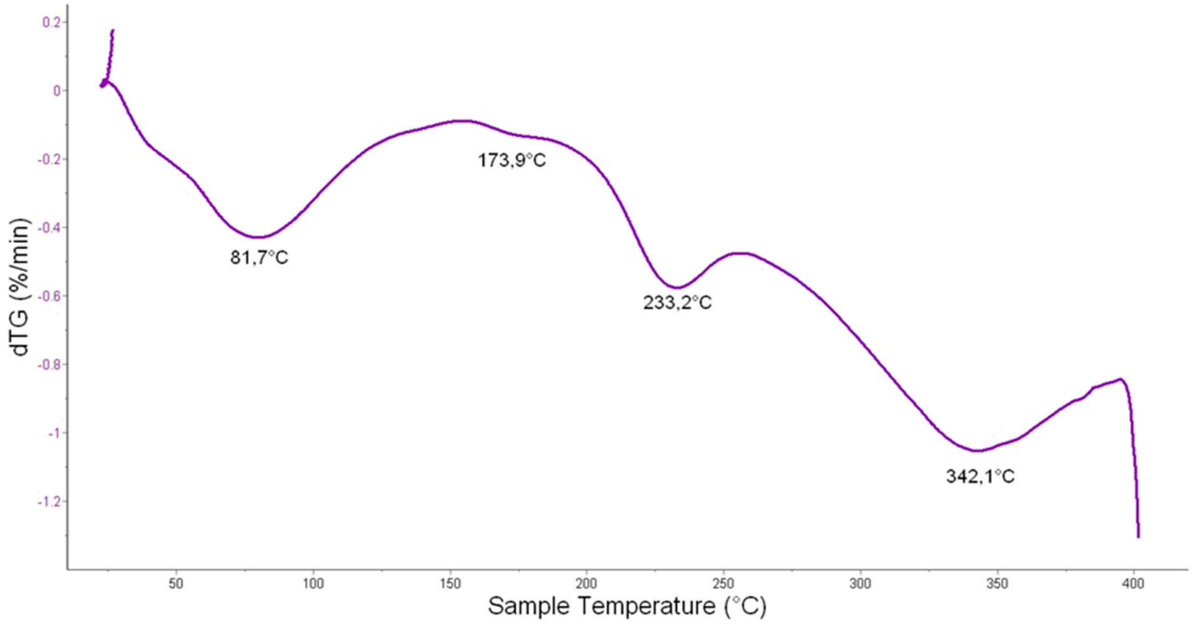

Figure S6. dTG curve test for LA.

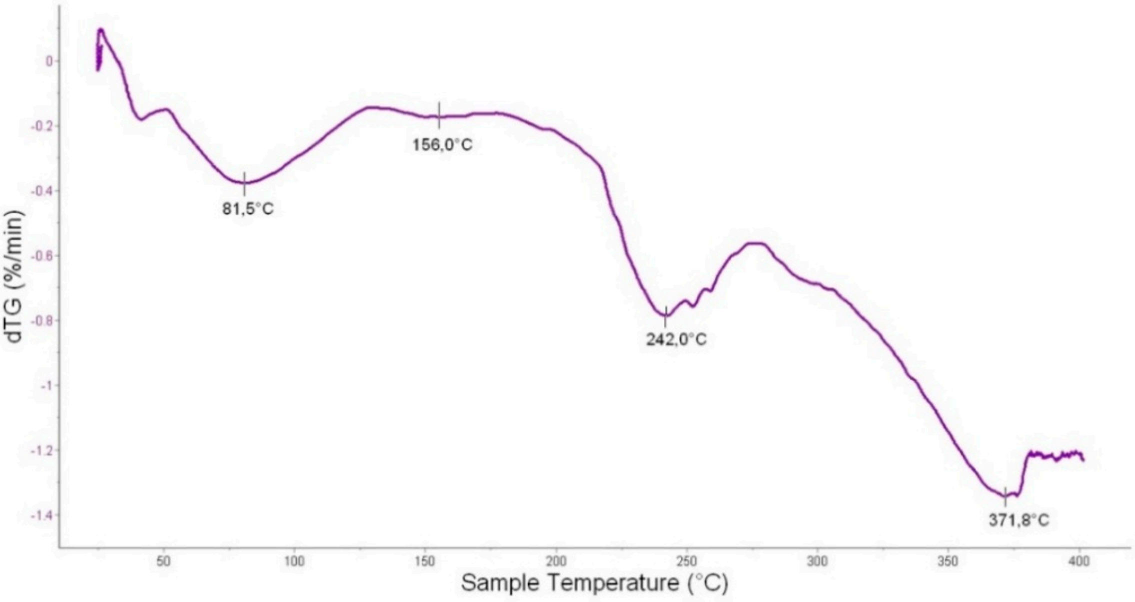

Figure S7. dTG curve test for LS.

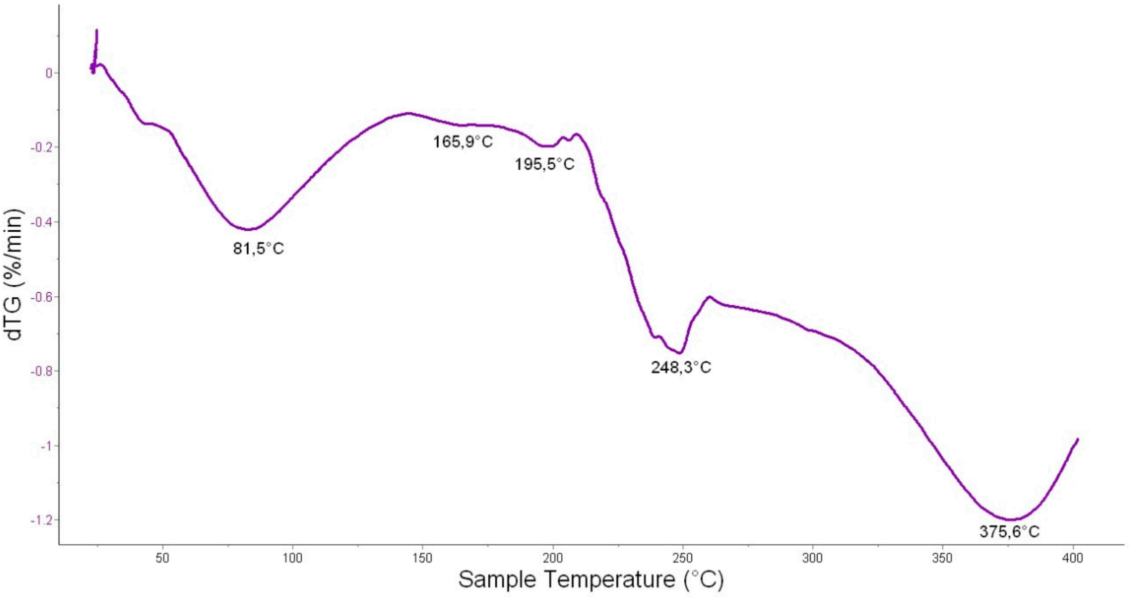

Figure S8. dTG curve test for LM.

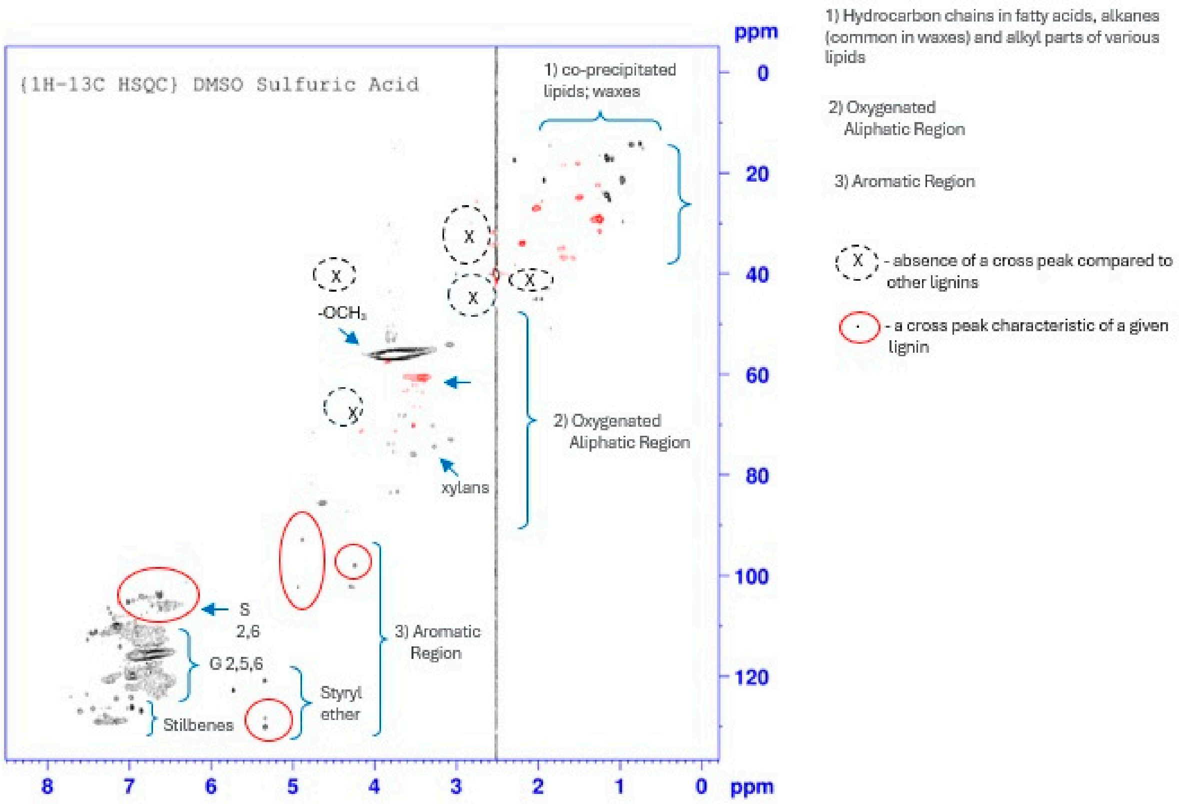

Figure S9. NMR spectrum of lignin LS.

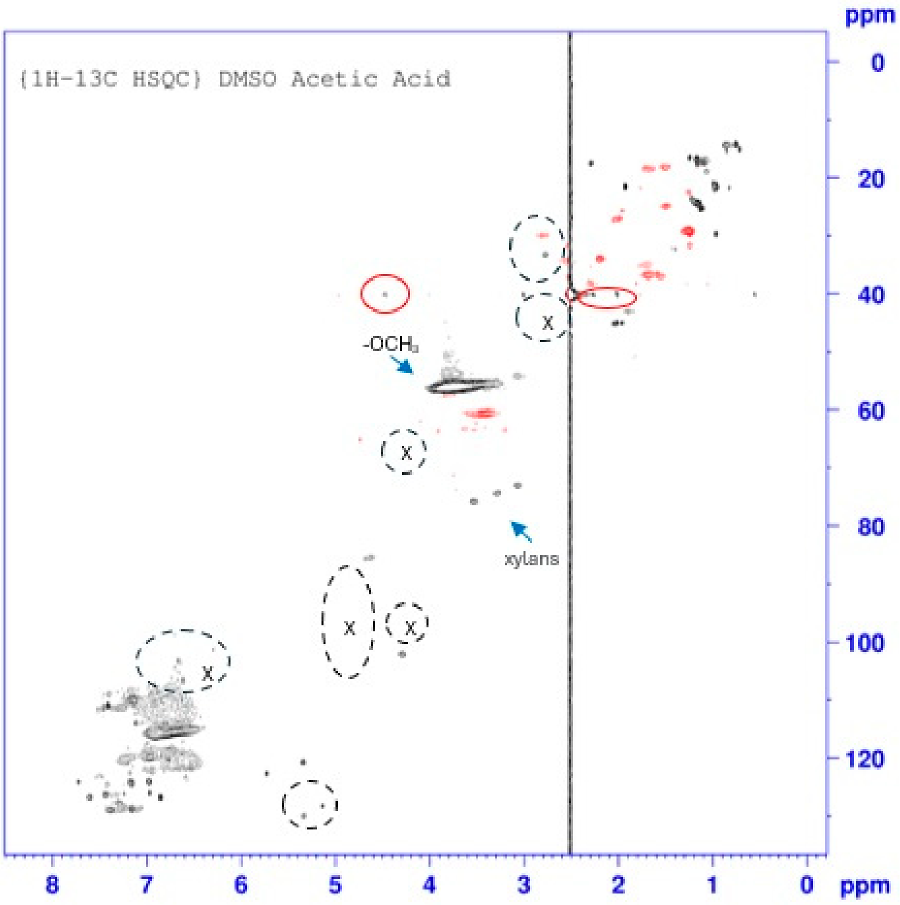

Figure S10. NMR spectrum of lignin LA.

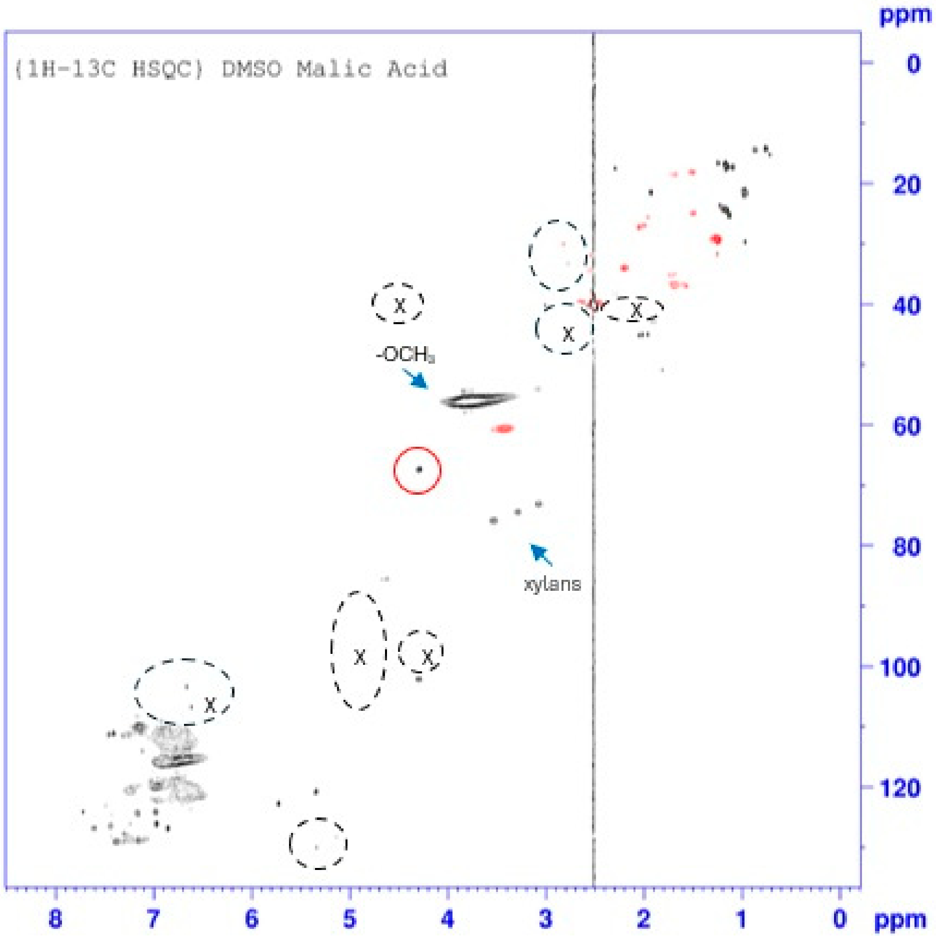

Figure S11. NMR spectrum of lignin LM.

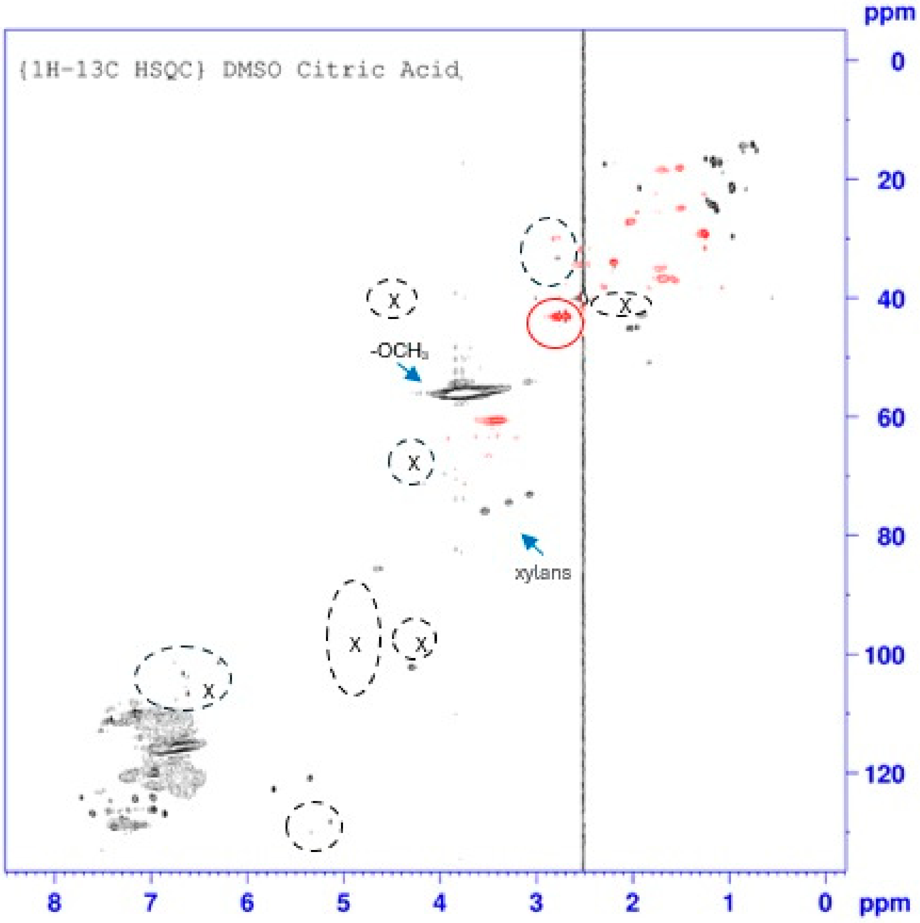

Figure S12. NMR spectrum of lignin LC.

LC

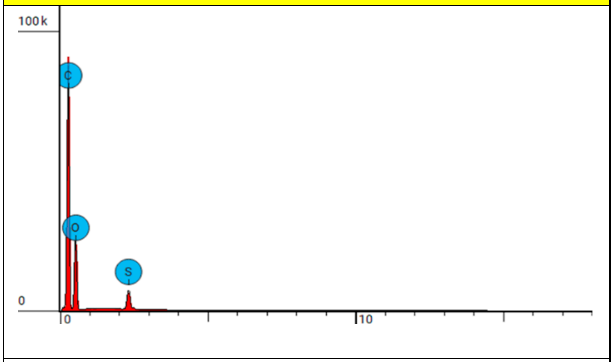

1 016 775 counts in 0:01:41 (9 992 c/s)

Element Symbol	Atomic Conc.	Weight Conc.
C	67.932	60.100
O	30.290	35.700
S	1.778	4.200

LA

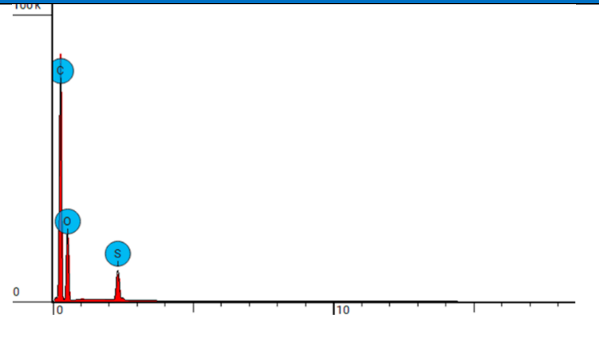

1 010 989 counts in 0:02:40 (6 309 c/s)

Element Symbol	Atomic Conc.	Weight Conc.
C	69.645	61.361
O	27.802	32.633
S	2.553	6.006

LM

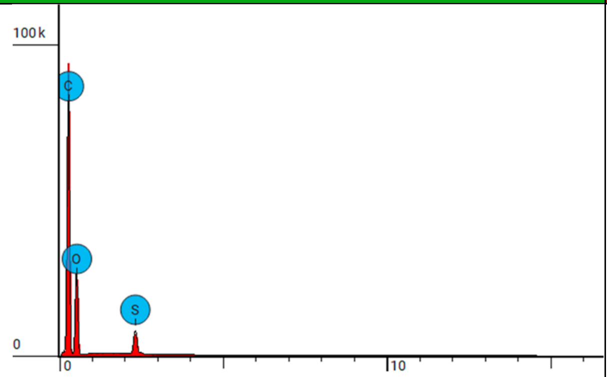

1 082 681 counts in 0:02:11 (8 202 c/s)

Element Symbol	Atomic Conc.	Weight Conc.
C	67.896	60.000
O	30.239	35.600
S	1.865	4.400

LS

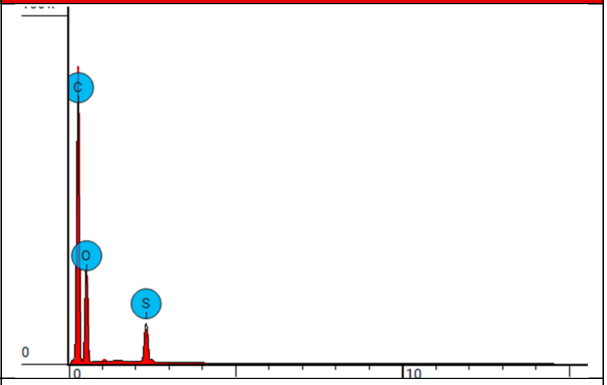

1 061 401 counts in 0:02:03 (8 565 c/s)

Element Symbol	Atomic Conc.	Weight Conc.
C	67.567	59.100
O	29.778	34.700
S	2.655	6.200

Figure S13. Elemental composition of lignin samples prior to adsorption, determined by Energy-Dispersive X-ray Spectroscopy (EDS). Spectra and quantitative results (atomic and weight concentration) are presented for lignins precipitated with citric (LC), acetic (LA), malic (LM), and sulfuric (LS) acids.

LC Cr

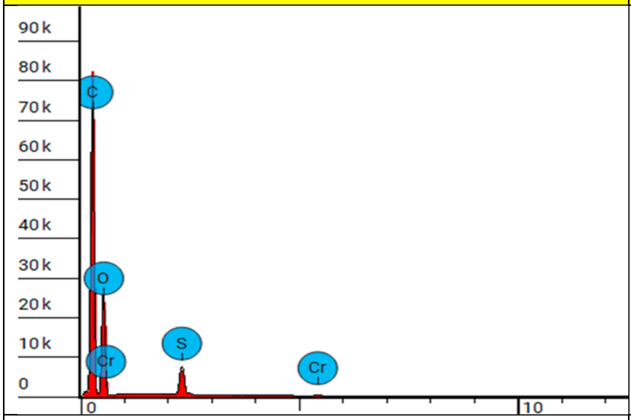

1 016 163 counts in 0:02:24 (7 052 c/s)

Element Symbol	Atomic Conc.	Weight Conc.
C	66.419	58.242
O	31.556	36.863
S	1.920	4.496
Cr	0.105	0.400

LC Cu

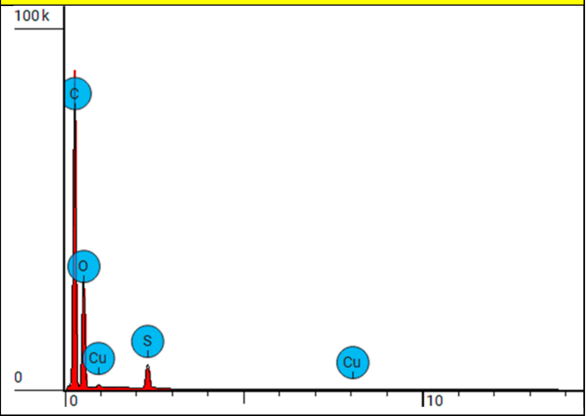

1 011 725 counts in 0:01:40 (10 042 c/s)

Element Symbol	Atomic Conc.	Weight Conc.
C	64.849	56.600
O	33.283	38.700
S	1.716	4.000
Cu	0.152	0.700

LC Ni

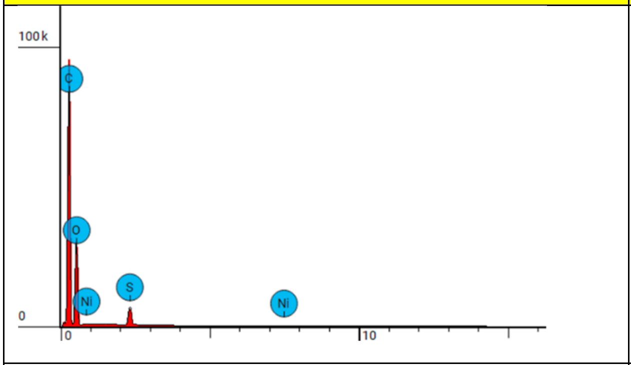

1 095 593 counts in 0:02:32 (7 200 c/s)

Element Symbol	Atomic Conc.	Weight Conc.
C	65.660	57.600
O	32.601	38.100
S	1.622	3.800
Ni	0.117	0.500

LC- Zn

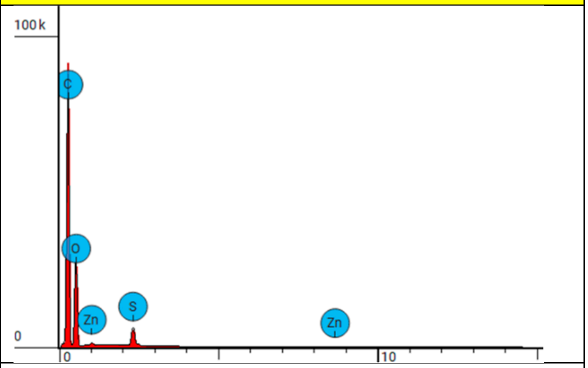

1 044 305 counts in 0:01:51 (9 338 c/s)

Element Symbol	Atomic Conc.	Weight Conc.
C	66.277	58.300
O	32.085	37.600
S	1.533	3.600
Zn	0.104	0.500

Figure S14. EDS analysis confirming the presence of heavy metals on citric acid lignin (LC) after the adsorption process.

LS Cr

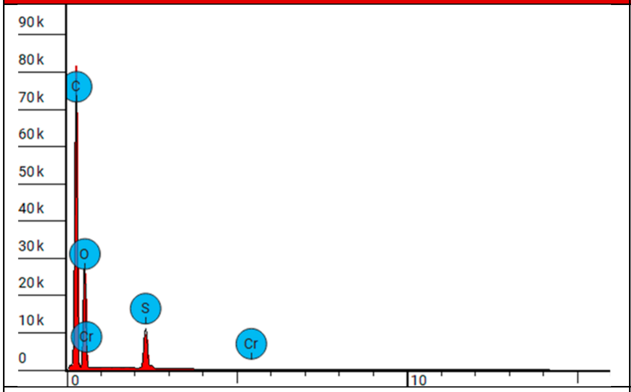

1 009 010 counts in 0:02:38 (6 378 c/s)

Element Symbol	Atomic Conc.	Weight Conc.
C	66.720	58.200
O	30.634	35.600
S	2.619	6.100
Cr	0.026	0.100

LS Cu

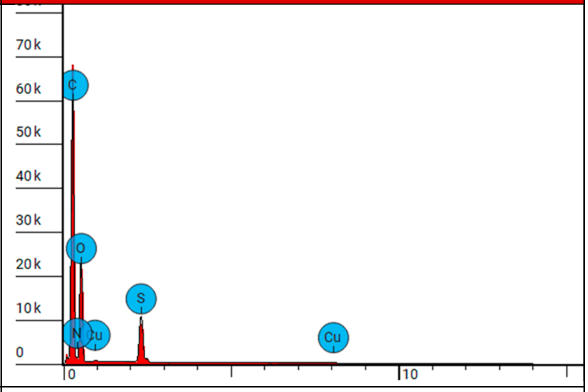

1 002 293 counts in 0:06:05 (2 739 c/s)

Element Symbol	Atomic Conc.	Weight Conc.
C	64.702	56.100
O	29.954	34.600
S	2.807	6.500
Cu	0.065	0.300

LS Ni

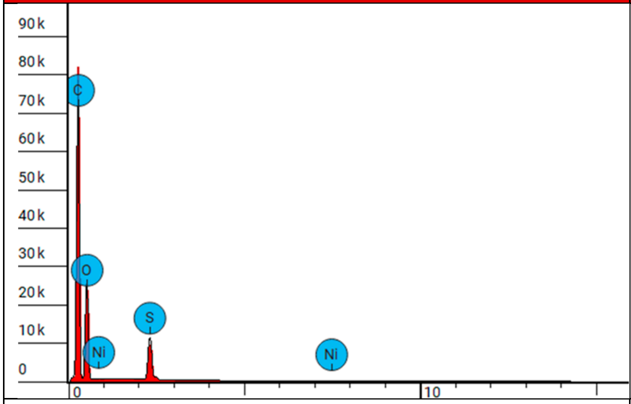

1 020 310 counts in 0:02:11 (7 730 c/s)

Element Symbol	Atomic Conc.	Weight Conc.
C	68.003	59.441
O	29.254	34.066
S	2.696	6.294
Ni	0.047	0.200

LS- Zn

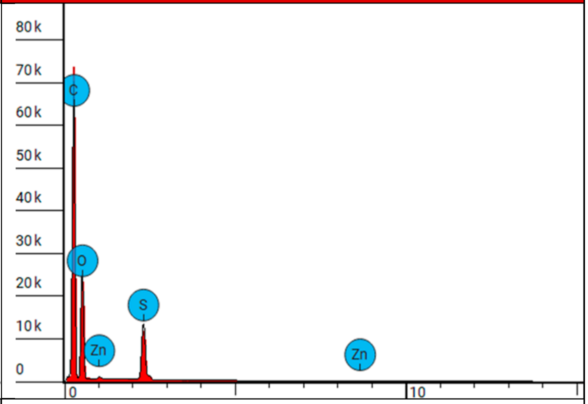

1 003 528 counts in 0:02:43 (6 148 c/s)

Element Symbol	Atomic Conc.	Weight Conc.
C	67.660	58.659
O	29.034	33.534
S	3.243	7.508
Zn	0.064	0.300

Figure S15. EDS analysis confirming the presence of heavy metals on sulfuric acid lignin (LS) after the adsorption process.

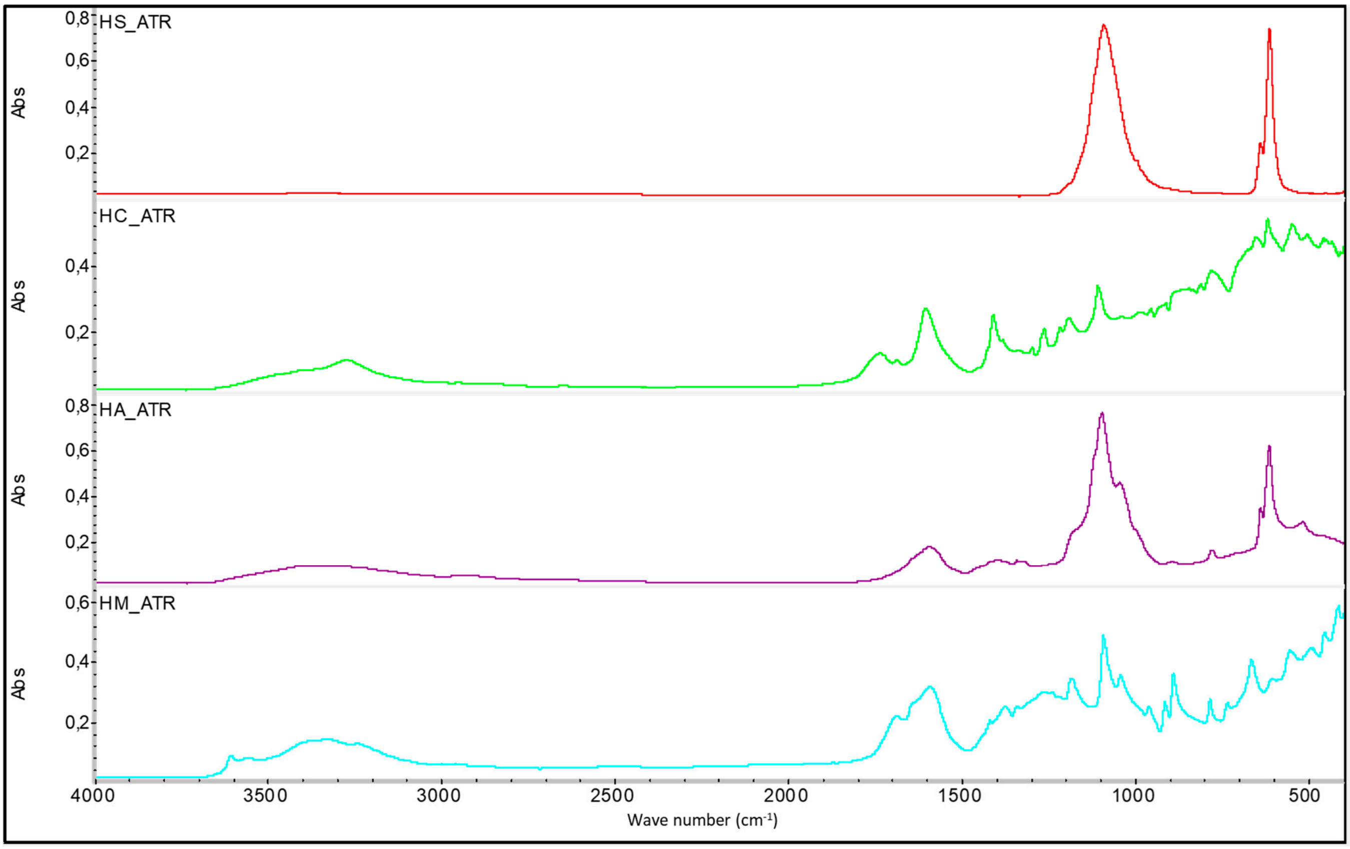

Figure S16. ATR spectrum of hemicellulose samples.

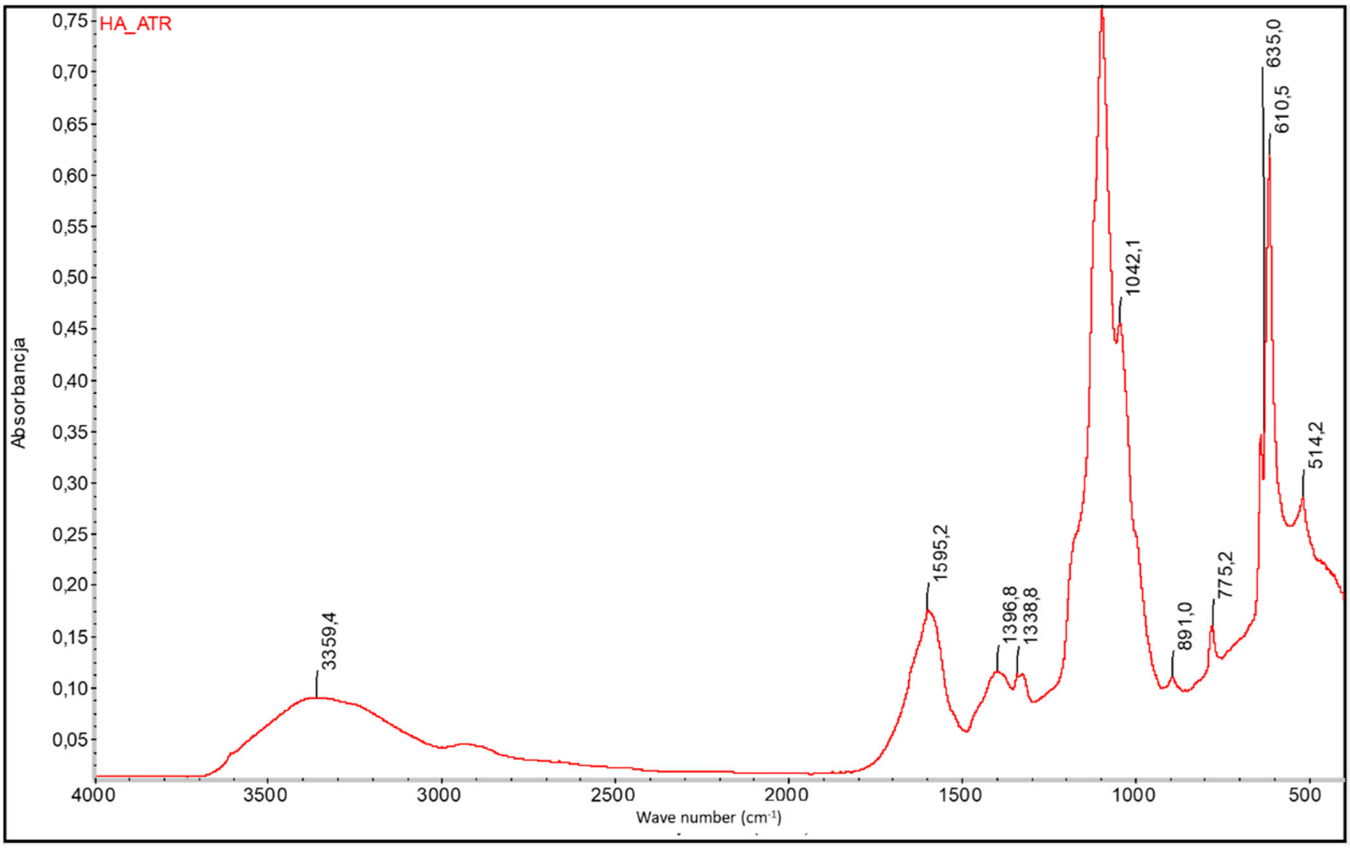

Figure S17. ATR spectrum of HA.

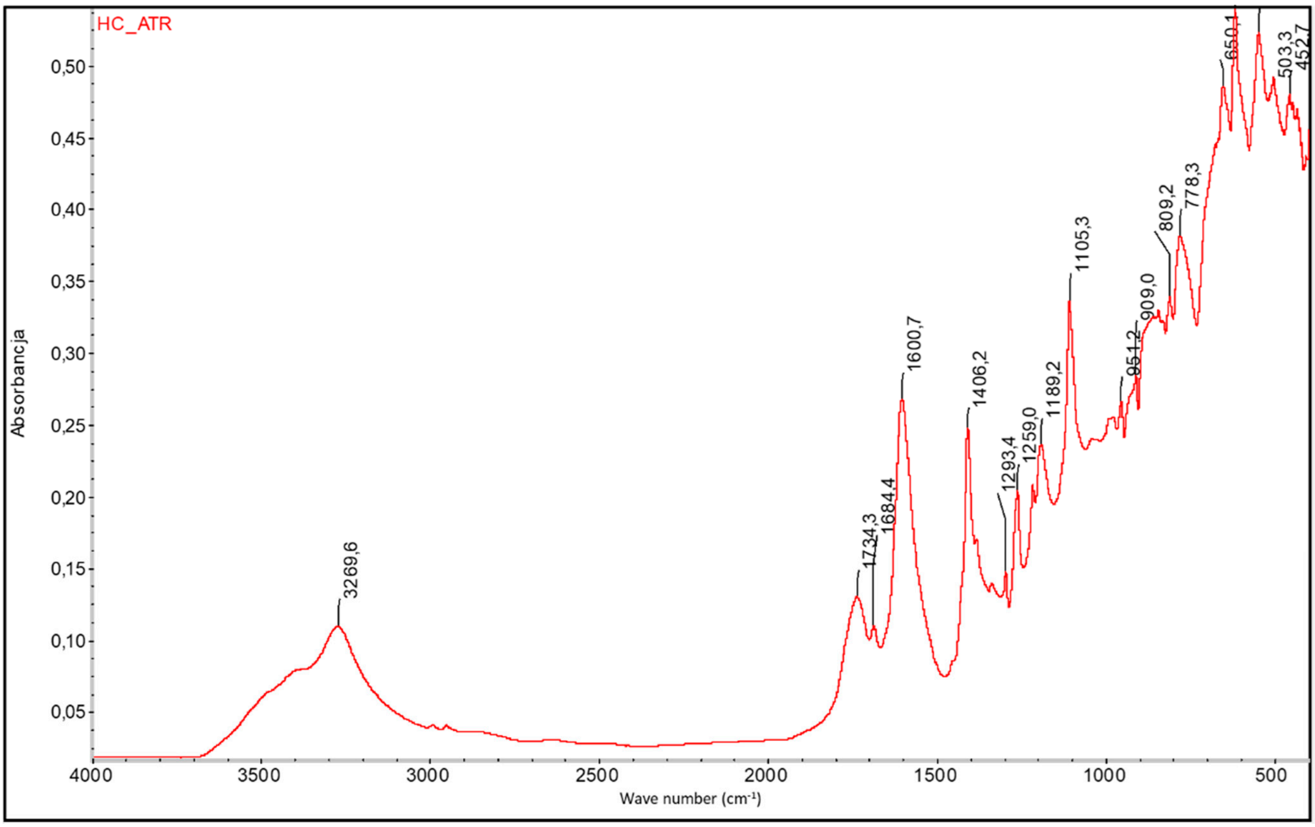

Figure S18. ATR spectrum of HC.

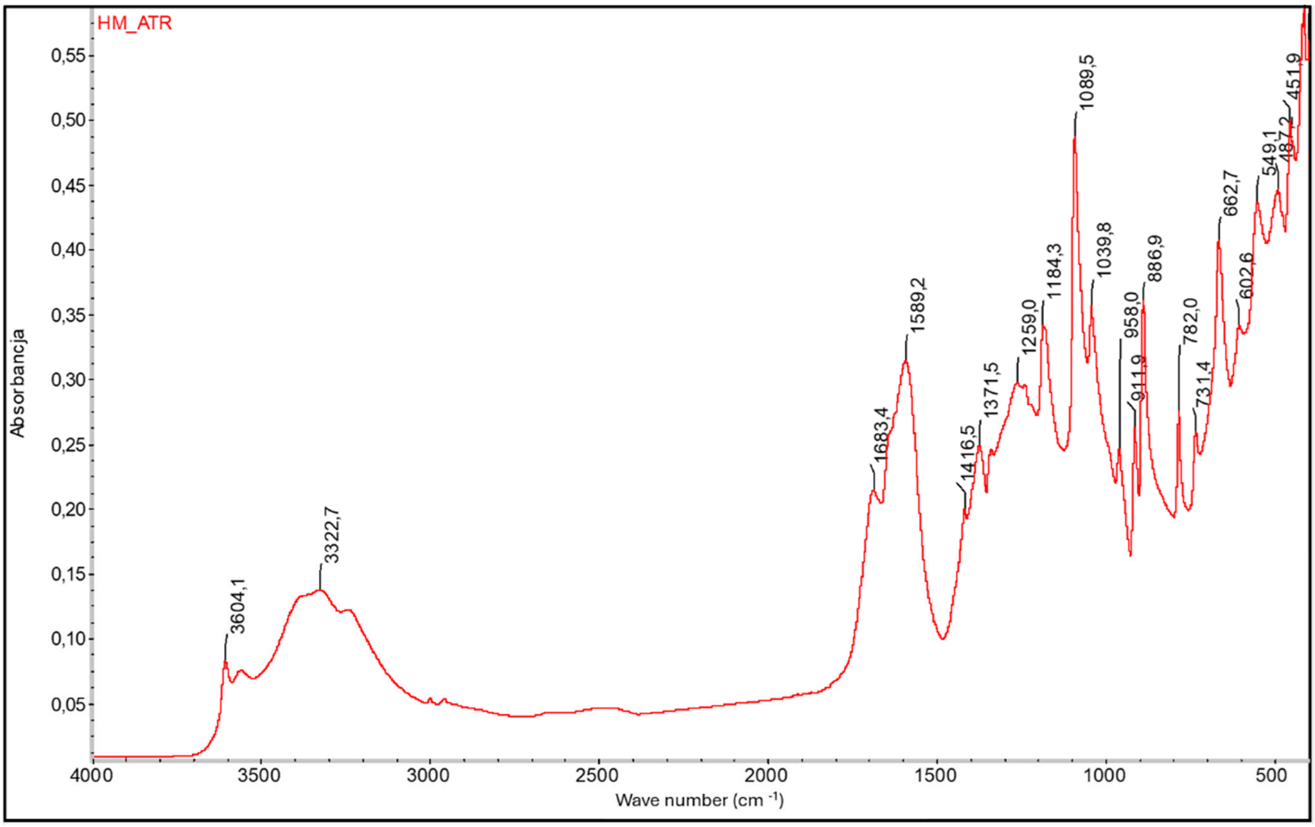

Figure S19. ATR spectrum of HM.

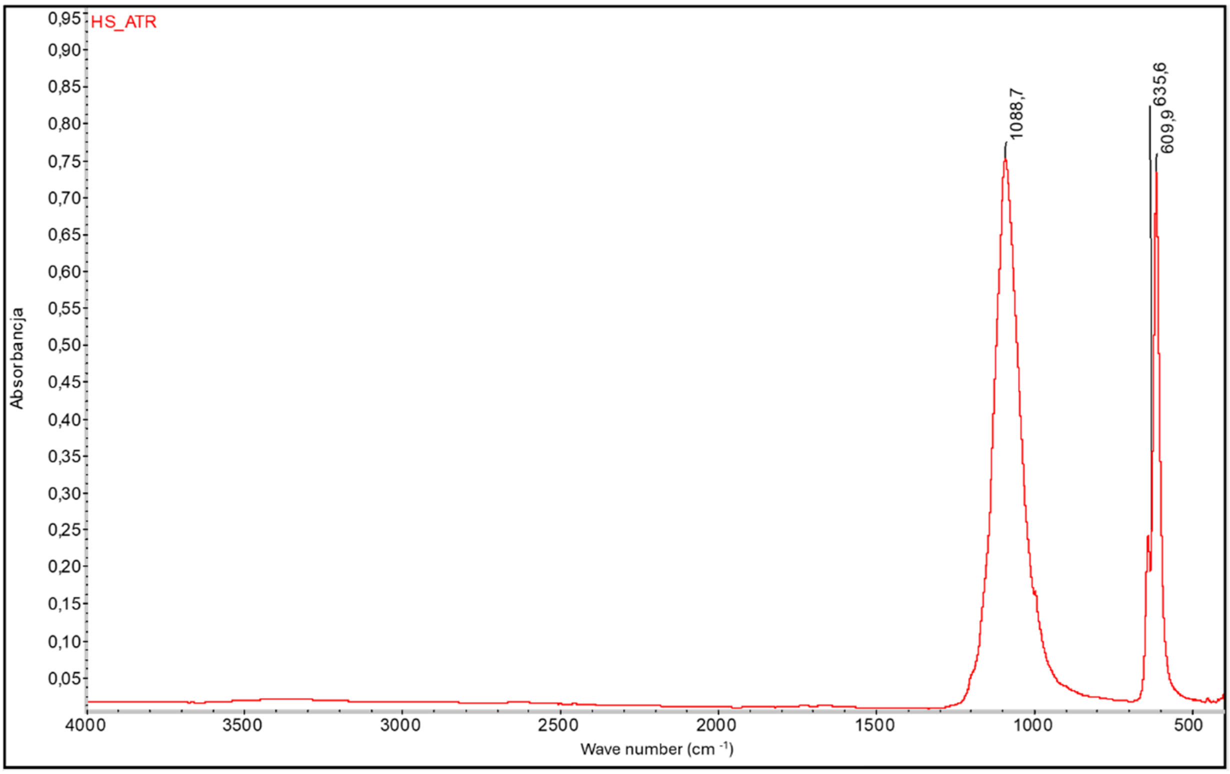

Figure S20. ATR spectrum of HS.

Table S2. EDS results for hemicellulose samples.

		**Atomic Concetration**			
	**Element Symbol**	**HS**	**HC**	**HA**	**HM**
	C	7.751	28.363	31.344	31.198
	O	55.460	56.672	48.125	57.026
	Na	21.444	12.483	11.829	11.042
	S	13.217	1.113	6.776	0.293
	K	2.128	1.369	1.926	0.441

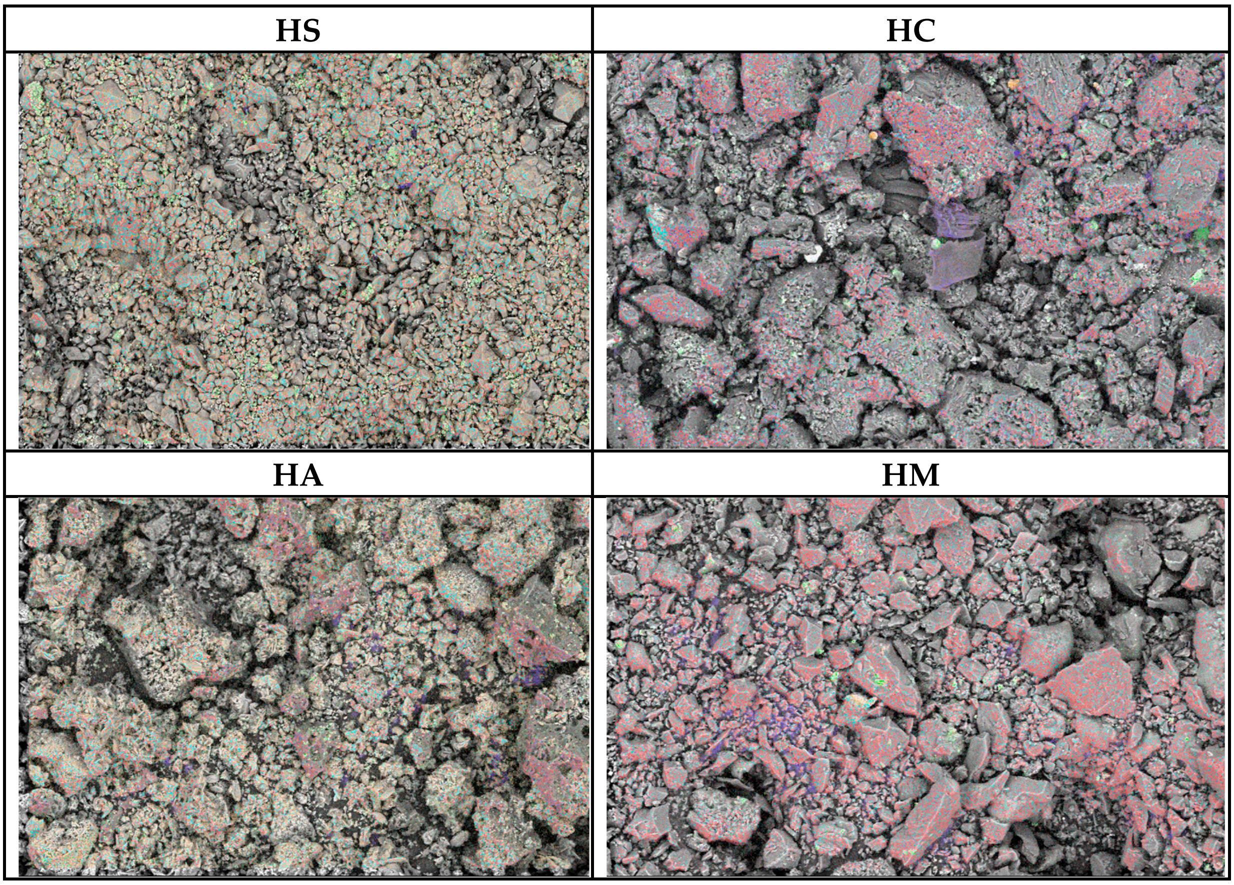

Figure S21. Photos obtained using the EDS method of hemicellulose samples.
